# Supplementary material for: Abnormal methylation in the NDUFA13 gene promoter of breast cancer cells breaks the cooperative DNA recognition by transcription factors
Source: QRB Discov. 2022 Nov 28;3:e23. doi: 10.1017/qrd.2022.21 (PMC10392677; doi:10.1017/qrd.2022.21)
Supplement: Supplementary file 1 [file S2633289222000217sup001.pdf]

Supporting Information

Abnormal methylation in NDUFA13 gene promoter of breast cancer cells breaks the cooperative DNA recognition by transcription factors

Hörberg Johanna, Hallbäck Björn, Moreau Kevin &_Reymer* Anna

Department of Chemistry and Molecular Biology, University of Gothenburg, 405 30 Göteborg, Sweden. * Email to: anna.reymer@gu.se

Supplementary methods

Protein-DNA contacts network analysis

Analysis of protein-DNA contacts for the E2F1-DP1-DNA/CEBPB-DNA/E2F1-DP1-CEBPB-DNA complexes is performed using CPPTRAJ16, for each trajectory snapshot extracted at 1 ps intervals. Protein-DNA contacts present for less than 10% of the trajectories are excluded. Protein-DNA contacts are characterized by pairs of residues, divided into 'specific', i.e., interactions formed between the protein side chains and DNA bases, and 'nonspecific', i.e., interactions formed with at least one of the backbones. The contacts formed between each protein-DNA residue pair are summed, where for simplicity, the contribution of each contact is set to 1. The distance limit of a hydrogen bond interaction is ≤ 4 Å between the relevant heavy atoms, and the angle limit is ≥ 135° at the intervening hydrogen atom. For a hydrophobic contact, the limit is ≤ 6Å between the centre of mass of hydrophobic residues (Ala, Ile, Leu, Met, Phe, Trp, and Cys) and DNA bases. The derived time series of protein-DNA interactions allow construction of dynamic contacts maps for specific and nonspecific contacts.

Supplementary figures

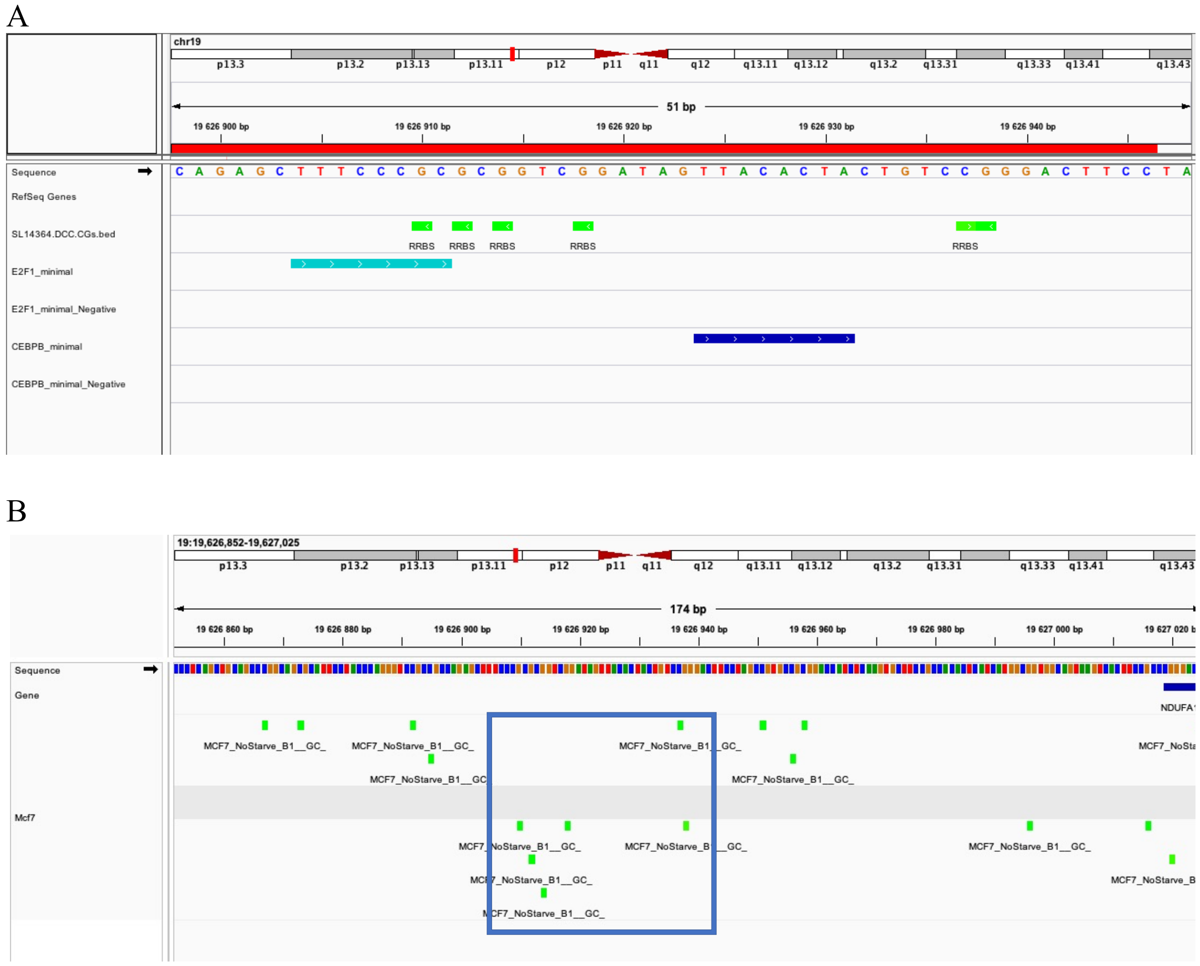

Figure S1. A: IGV (Integrative Genome Viewer) screenshot depicting the studied wild-type DNA sequence with the cytosine methylation marks detected in the promoter of NDUFA13 gene of breast cancer cell line MCF7; binding sites of two collaborative transcription factor dimers E2F1-DP1 and CEBPB. **B:** IGV screenshot depicting all identified CpG methylation sites (14 in total) within the promoter of NDUFA13 gene of breast cancer cell line MCF7. The studied six CpG methylation marks are enclosed by a blue rectangle.

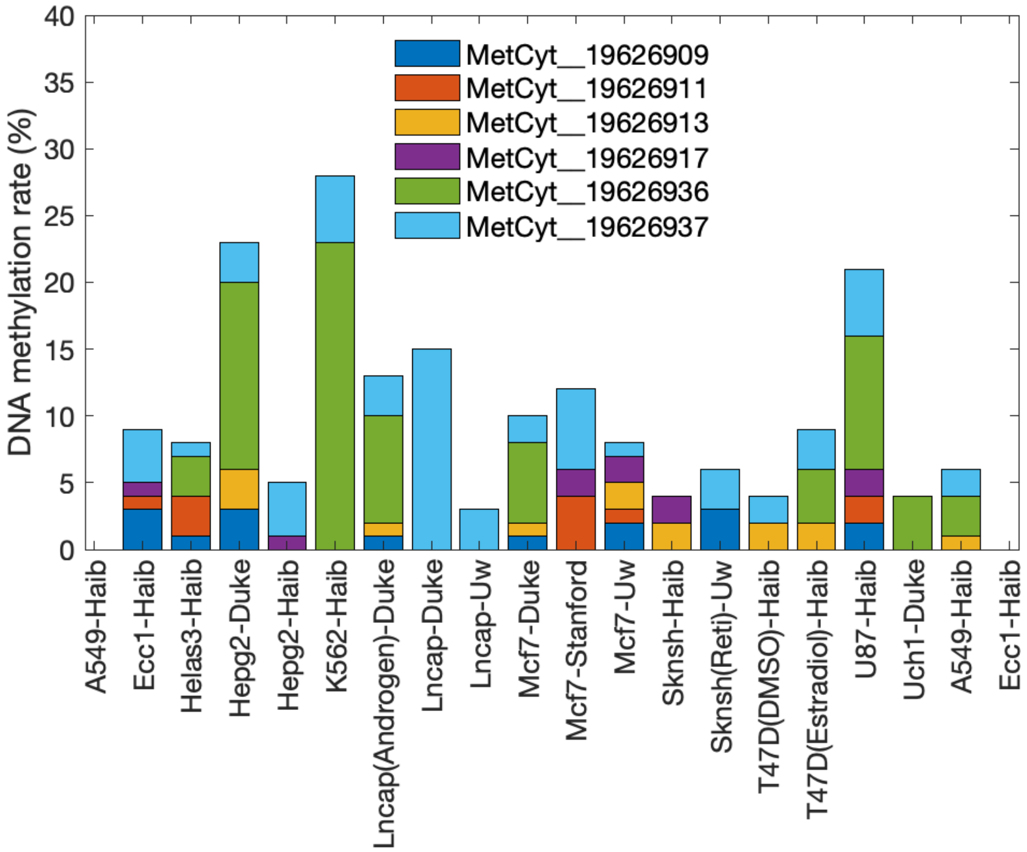

Figure S2: DNA methylation rates (percentage of DNA molecules that exhibit cytosine methylation at a specific CpG site**)** of available different cancerous cell lines within the studied region of NDUFA-13 promoter at the 19th chromosome, position: 19626898-19626946. The description of cell lines codes can be found in table S1 below. The DNA methylation data were obtained through assays with a modified version of Reduced Representation Bisulfite Sequencing. (1) The data and the protocol for their production, used to construct the figure and further details can be found at: http://genome.ucsc.edu/cgi-bin/hgFileUi?db=hg19&g=wgEncodeHaibMethylRrbs

Table S1: Cell line codes as used in Figure S2, with the respective descriptions of the cell lines. The cell line codes are given in the format "code" – "source/lab". For the "source/lab" the following abbreviations are used: "Haib", "Duke", "UW", "Stanford". For further details see: http://genome.ucsc.edu/cgi-bin/hgFileUi?db=hg19&g=wgEncodeHaibMethylRrbs

Cell line Code	Cell line description
A549-Haib	Human epithelial cell line from lung carcinoma
Ecc1-Haib	Human endometrial cell adenocarcinoma
Helas3-Haib	Human cervical carcinoma
Hepg2-Duke	Human liver carcinoma
Hepg2-Haib	Human liver carcinoma
K562-Haib	Human leukemia
Lncap(Androgen)-Duke	Human prostate adenocarcinoma treated with androgen
Lncap-Duke	Human prostate adenocarcinoma
Lncap-Uw	Human prostate adenocarcinoma
Mcf7-Duke	Human breast, mammary gland, adenocarcinoma
Mcf7-Stanford	Human breast, mammary gland, adenocarcinoma
Mcf7-Uw	Human breast, mammary gland, adenocarcinoma
Sknsh-Haib	Human neuroblastoma
Sknsh(Reti)-Uw	Human neuroblastoma, treated with retinol
T47D(DMSO)-Haib	Human epithelial cell line from mammary ductal carcinoma treated with DMSO 0.02% for 24hr
T47D(Estradiol)-Haib	Human epithelial cell line from mammary ductal carcinoma treated with estradiol 10nM for 24hr
U87-Haib	Human cell line with epithelial morphology from malignant gliomas from a patient, likely, with glioblastoma
Uch1-Duke	Human chordoma cell line

A

```
E2F1     121 SPGEKSRYETSLNLTTKRFLELLSHSADGVVDLNWAAEVLKV-QKRRIYD
             .||..||:|.||.|.|.:|:.||..:.|||:||..||:.|.| |||||||
E2F4      11 PPGTPSRHEKSLGLLTTKFVSLLQEAKDGVLDLKLAADTLAVRQKRRIYD

E2F1         ITNVLEGIQLIAKKSKNHIQWLGSH 194
             ||||||||.||.|||||.|||.|..
E2F4         ITNVLEGIGLIEKKSKNSIQWKGVG 85
```

B

```
DP1      113 GKGLRHFSMKVCEKVQRKGTTSYNEVADELVAEFSAADNHILPNESAYDQ
             |||||||||||||||||||||||||||||||:||:.::|| |..:|||||
DP2      129 GKGLRHFSMKVCEKVQRKGTTSYNEVADELVSEFTNSNNH-LAADSAYDQ

DP1          KNIRRRVYDALNVLMAMNIISKEKKEIKWIGLP 195
             ||||||||||||||||||||||||||||||||
DP2          KNIRRRVYDALNVLMAMNIISKEKKEIKWIGLP 210
```

Figure S3: Pairwise sequence alignment used for homology modelling of the E2F1-DP1 heterodimer. **A.** Alignment between E2F1 (Query) and E2F4 (Template). **B.** Alignment between DP1 (Query) and DP2 (Template).

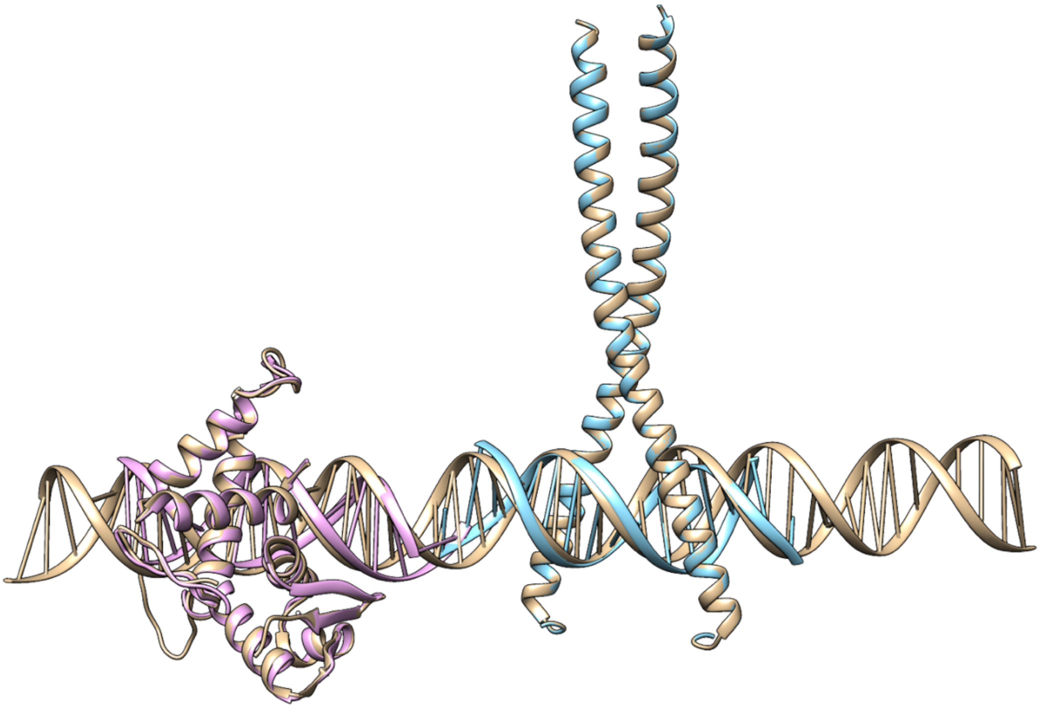

Figure S4: E2F4-DP2 (pink) and 1GU4 (cyan) PDB crystal structures superposed on the complete E2F1-DP1-CEBPB molecular model after homology modelling and docking to the DNA.

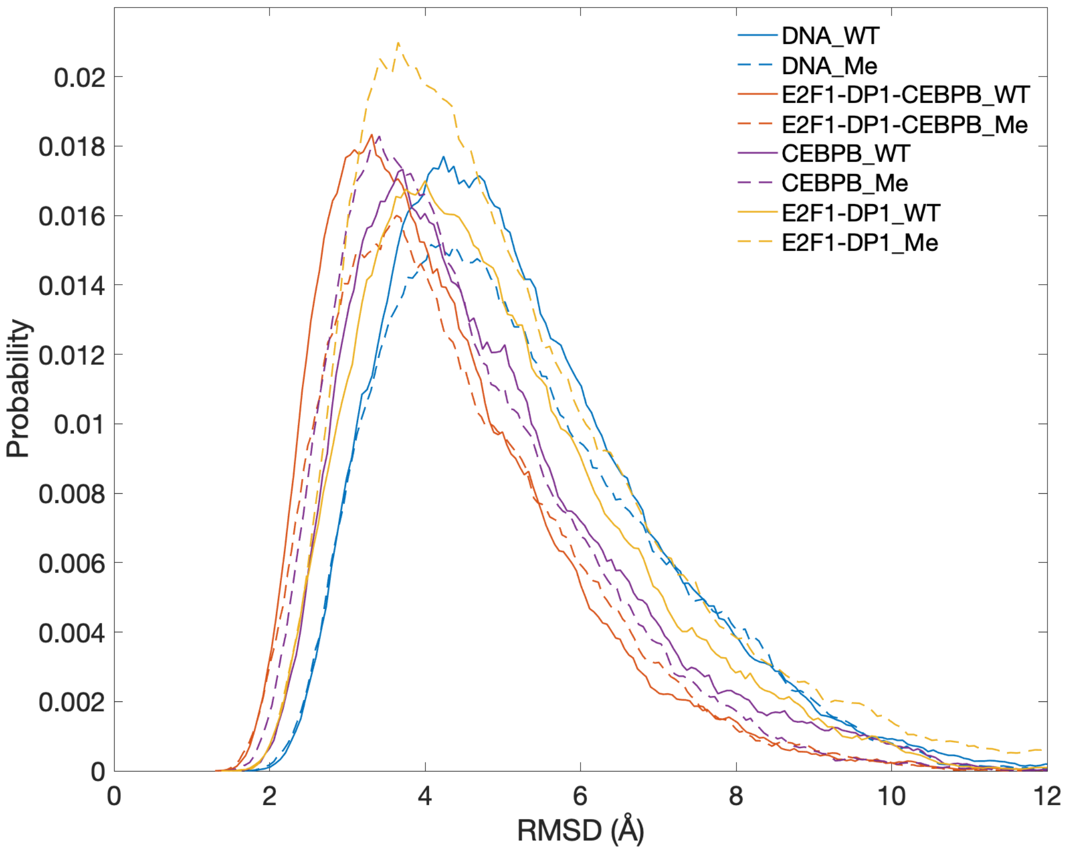

Figure S5: RMSD Distributions for the heavy atoms of DNA in different simulated systems.

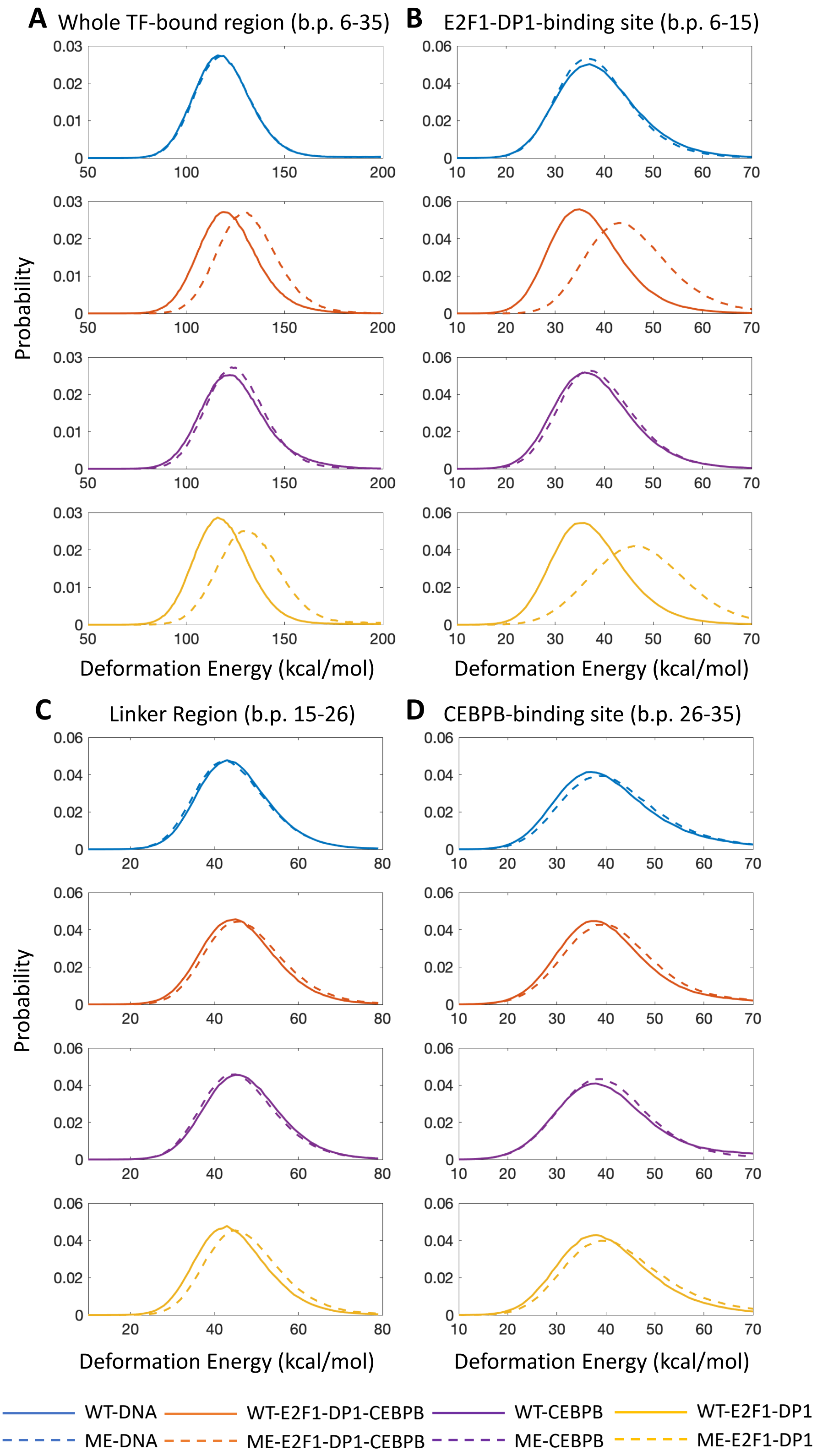

Figure S6: Distribution of DNA deformation energies calculated for the entire trajectories (excluding first 100ns of equilibration) with a multivariate Ising model (2) for **A.** Whole TF-bound region (b.p. 6-35), which includes both response elements and the adjacent flanking nucleotides, **B.** E2F1-DP1-binding site (b.p. 6-15), **C.** Linker-region (b.p. 15-26) and **D.** CEBPB-binding site (b.p. 26-35). WT-systems are denoted with solid bold lines and ME-systems are denoted with dashed lines.

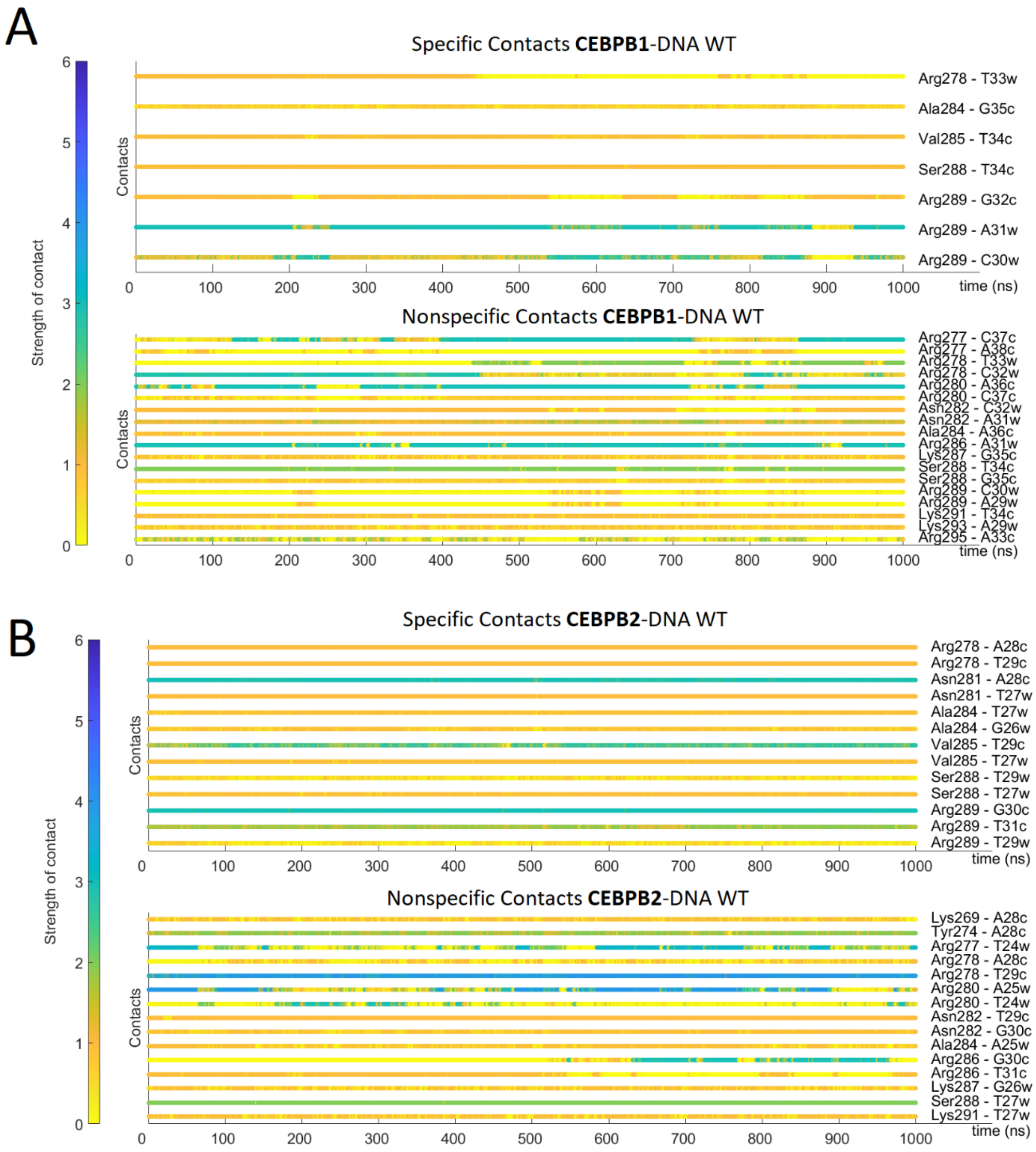
A
Specific Contacts CEBPB1-DNA WT
Strength of contact
Contacts
Arg278 - T33w
Ala284 - G35c
Val285 - T34c
Ser288 - T34c
Arg289 - G32c
Arg289 - A31w
Arg289 - C30w
time (ns)
Nonspecific Contacts CEBPB1-DNA WT
Arg277 - C37c
Arg277 - A38c
Arg278 - T33w
Arg278 - C32w
Arg280 - A36c
Arg280 - C37c
Asn282 - C32w
Asn282 - A31w
Ala284 - A36c
Arg286 - A31w
Lys287 - G35c
Ser288 - T34c
Ser288 - G35c
Arg289 - C30w
Arg289 - A29w
Lys291 - T34c
Lys293 - A29w
Arg295 - A33c
B
Specific Contacts CEBPB2-DNA WT
Arg278 - A28c
Arg278 - T29c
Asn281 - A28c
Asn281 - T27w
Ala284 - T27w
Ala284 - G26w
Val285 - T29c
Val285 - T27w
Ser288 - T29w
Ser288 - T27w
Arg289 - G30c
Arg289 - T31c
Arg289 - T29w
Nonspecific Contacts CEBPB2-DNA WT
Lys269 - A28c
Tyr274 - A28c
Arg277 - T24w
Arg278 - A28c
Arg278 - T29c
Arg280 - A25w
Arg280 - T24w
Asn282 - T29c
Asn282 - G30c
Ala284 - A25w
Arg286 - G30c
Arg286 - T31c
Lys287 - G26w
Ser288 - T27w
Lys291 - T27w

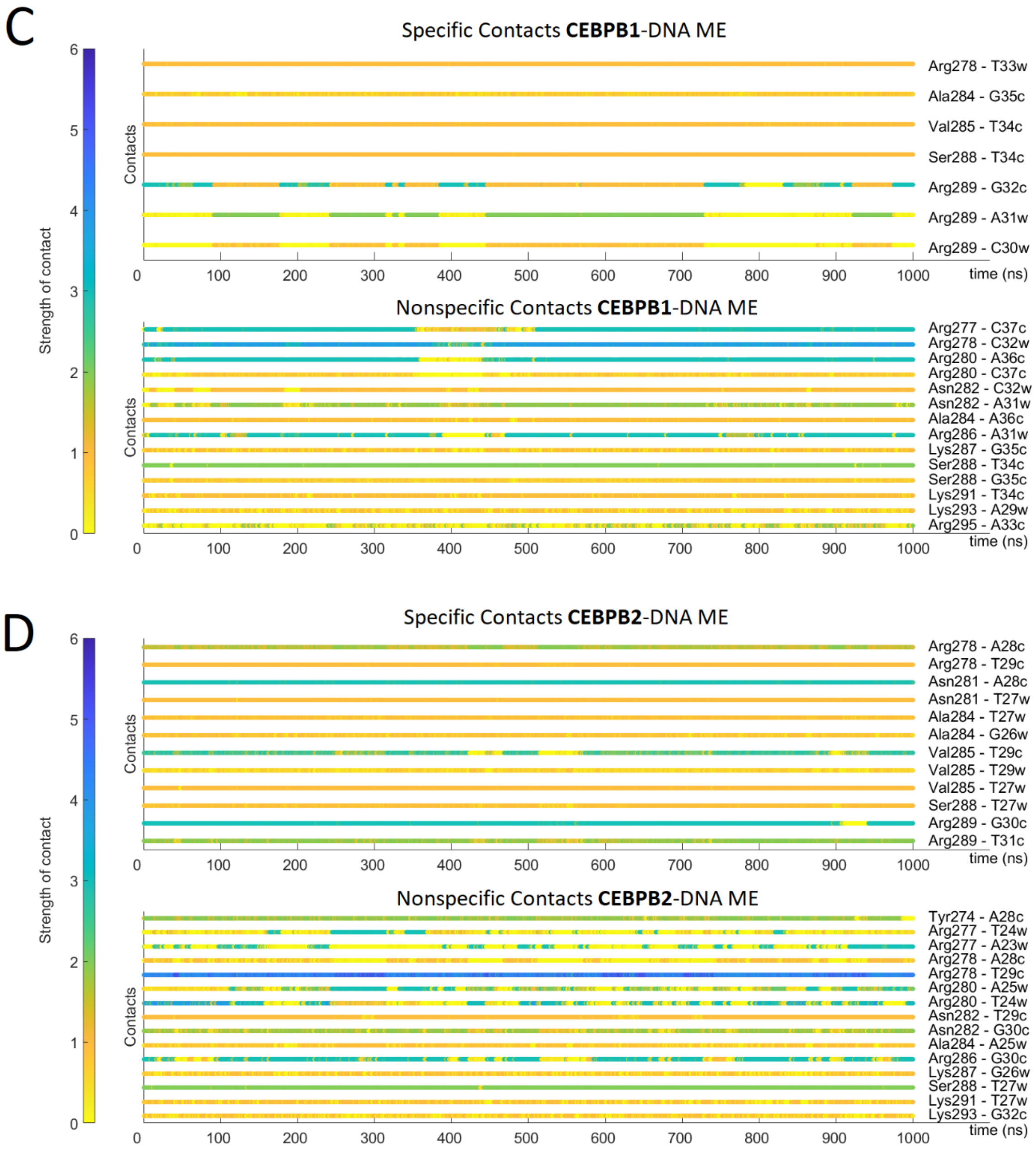

Figure S7. Time-evolution of the specific and nonspecific protein-DNA contacts for the CEBPB-DNA system, CEBPB1 and CEBPB2 correspond to different monomers: **A:** WT CEBPB1-DNA; **B:** WT CEBPB2-DNA; **C:** ME CEBPB1-DNA; **D:** ME CEBPB2-DNA.

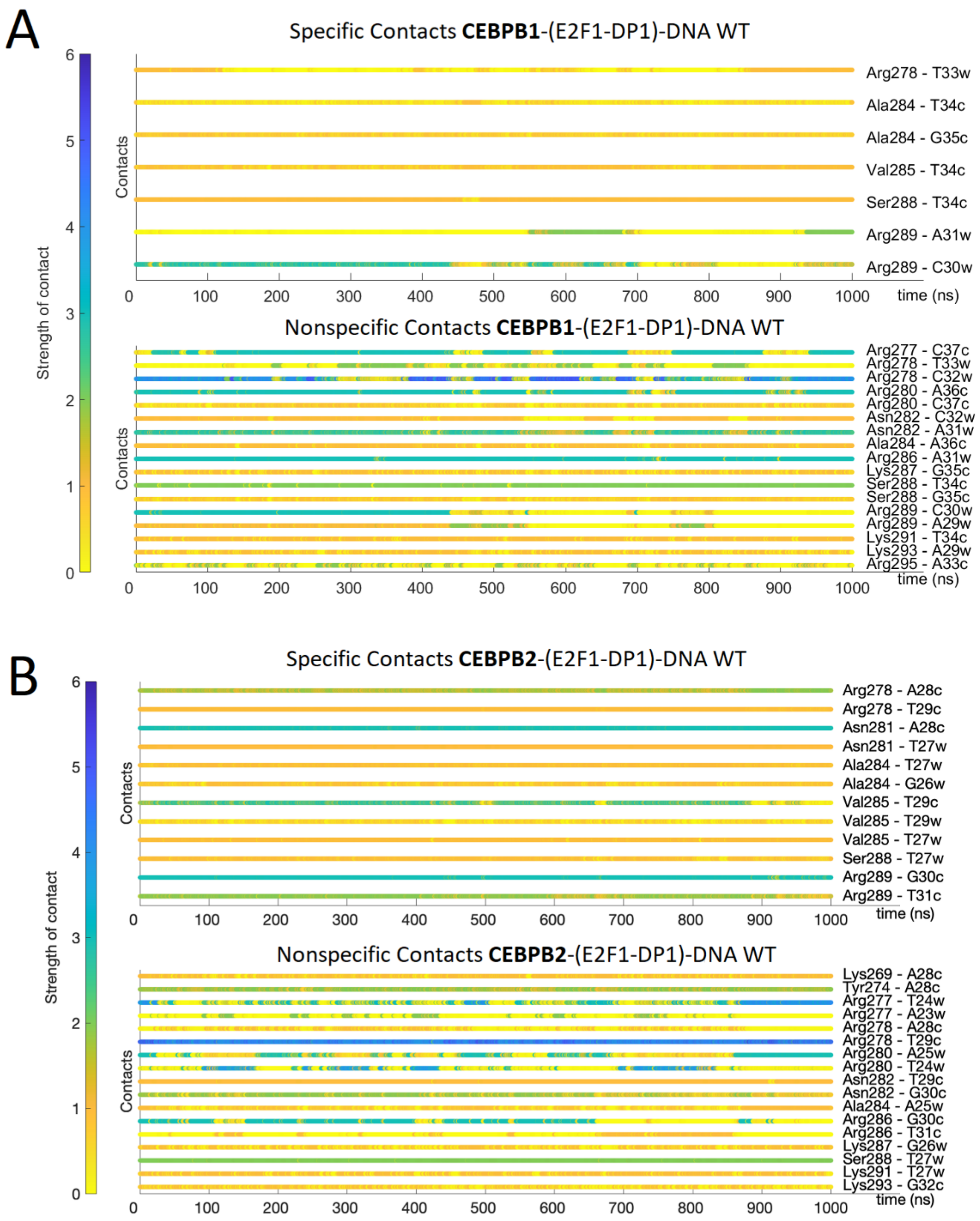
A
Specific Contacts CEBPB1-(E2F1-DP1)-DNA WT
Arg278 - T33w
Ala284 - T34c
Ala284 - G35c
Val285 - T34c
Ser288 - T34c
Arg289 - A31w
Arg289 - C30w
Contacts
time (ns)
Strength of contact
Nonspecific Contacts CEBPB1-(E2F1-DP1)-DNA WT
Arg277 - C37c
Arg278 - T33w
Arg278 - C32w
Arg280 - A36c
Arg280 - C37c
Asn282 - C32w
Asn282 - A31w
Ala284 - A36c
Arg286 - A31w
Lys287 - G35c
Ser288 - T34c
Ser288 - G35c
Arg289 - C30w
Arg289 - A29w
Lys291 - T34c
Lys293 - A29w
Arg295 - A33c
B
Specific Contacts CEBPB2-(E2F1-DP1)-DNA WT
Arg278 - A28c
Arg278 - T29c
Asn281 - A28c
Asn281 - T27w
Ala284 - T27w
Ala284 - G26w
Val285 - T29c
Val285 - T29w
Val285 - T27w
Ser288 - T27w
Arg289 - G30c
Arg289 - T31c
Nonspecific Contacts CEBPB2-(E2F1-DP1)-DNA WT
Lys269 - A28c
Tyr274 - A28c
Arg277 - T24w
Arg277 - A23w
Arg278 - A28c
Arg278 - T29c
Arg280 - A25w
Arg280 - T24w
Asn282 - T29c
Asn282 - G30c
Ala284 - A25w
Arg286 - G30c
Arg286 - T31c
Lys287 - G26w
Ser288 - T27w
Lys291 - T27w
Lys293 - G32c

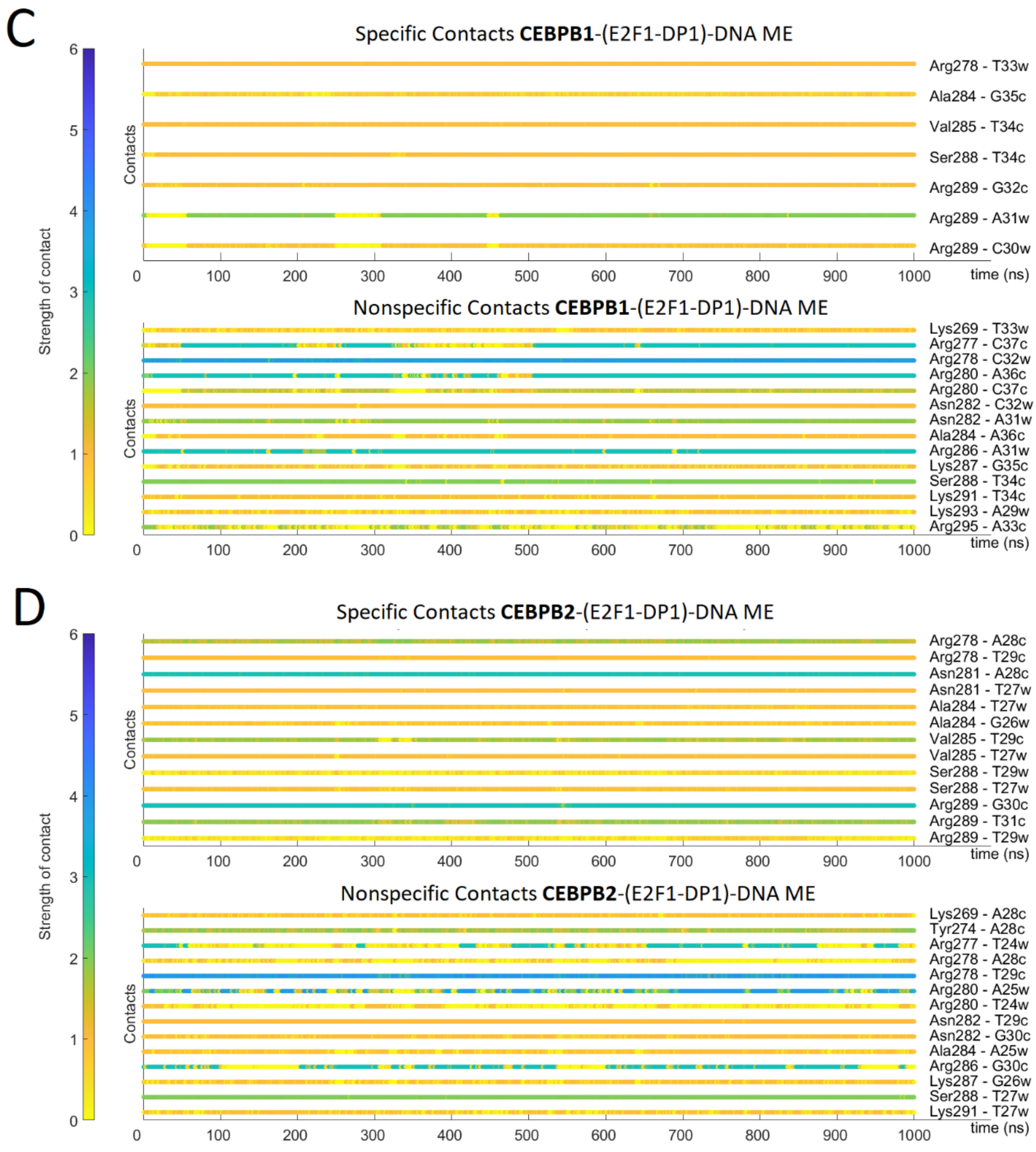

Figure S8. Time-evolution of the specific and nonspecific protein-DNA contacts for the E2F1-DP1-CEBPB-DNA system: **A:** WT CEBPB1-DNA; **B:** WT CEBPB2-DNA; **C:** ME CEBPB1-DNA; **D:** ME CEBPB2-DNA.

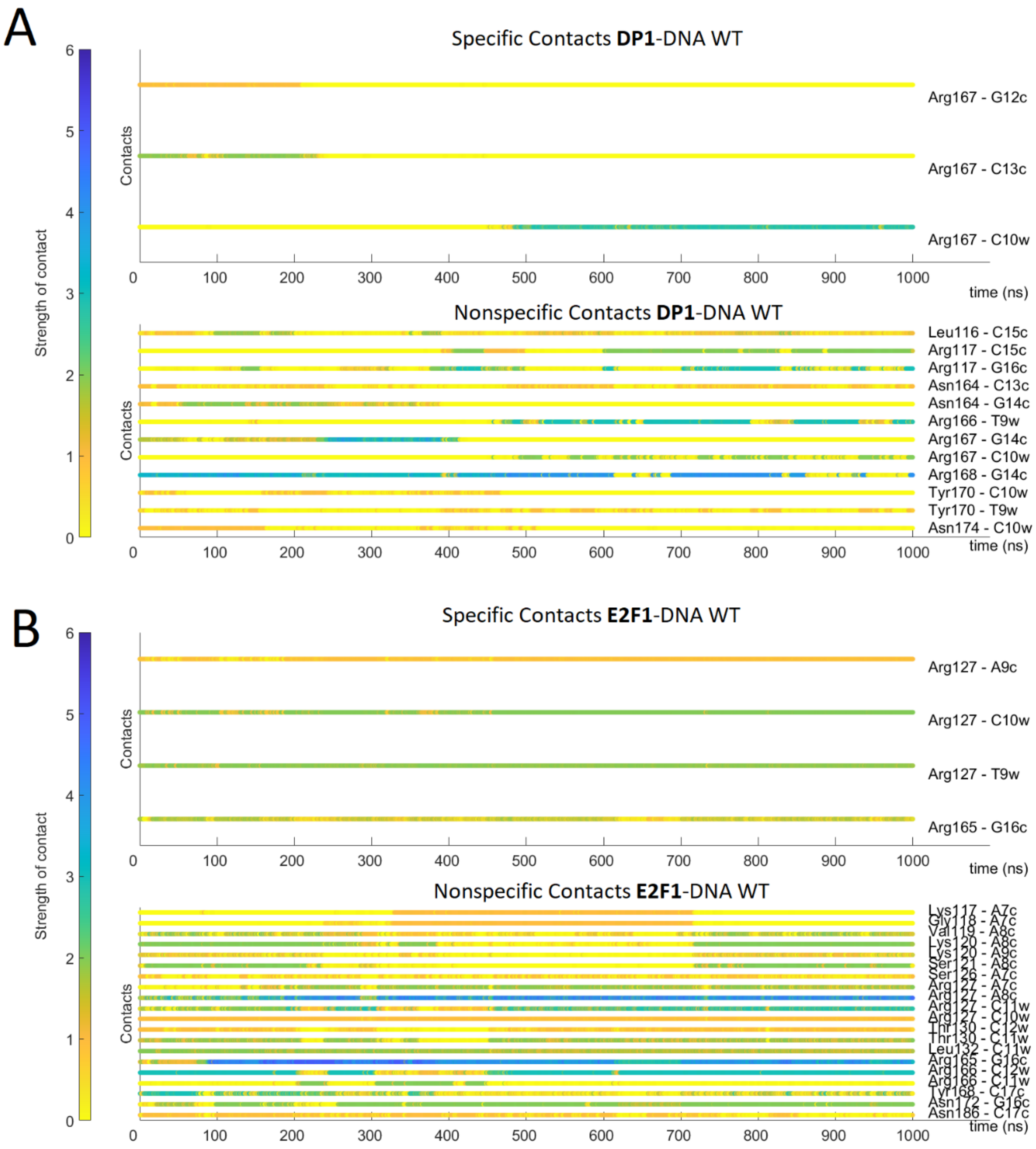
A
Specific Contacts DP1-DNA WT
Strength of contact
Contacts
Arg167 - G12c
Arg167 - C13c
Arg167 - C10w
time (ns)
Nonspecific Contacts DP1-DNA WT
Leu116 - C15c
Arg117 - C15c
Arg117 - G16c
Asn164 - C13c
Asn164 - G14c
Arg166 - T9w
Arg167 - G14c
Arg167 - C10w
Arg168 - G14c
Tyr170 - C10w
Tyr170 - T9w
Asn174 - C10w
B
Specific Contacts E2F1-DNA WT
Arg127 - A9c
Arg127 - C10w
Arg127 - T9w
Arg165 - G16c
Nonspecific Contacts E2F1-DNA WT
Lys117 - A7c
Gly118 - A7c
Val119 - A8c
Lys120 - A8c
Lys120 - A9c
Ser121 - A8c
Ser126 - A7c
Arg127 - A7c
Arg127 - A8c
Arg127 - C11w
Arg127 - C10w
Thr130 - C12w
Thr130 - C11w
Leu132 - C11w
Arg165 - G16c
Arg166 - C12w
Arg166 - C11w
Tyr168 - C17c
Asn172 - G16c
Asn186 - C17c

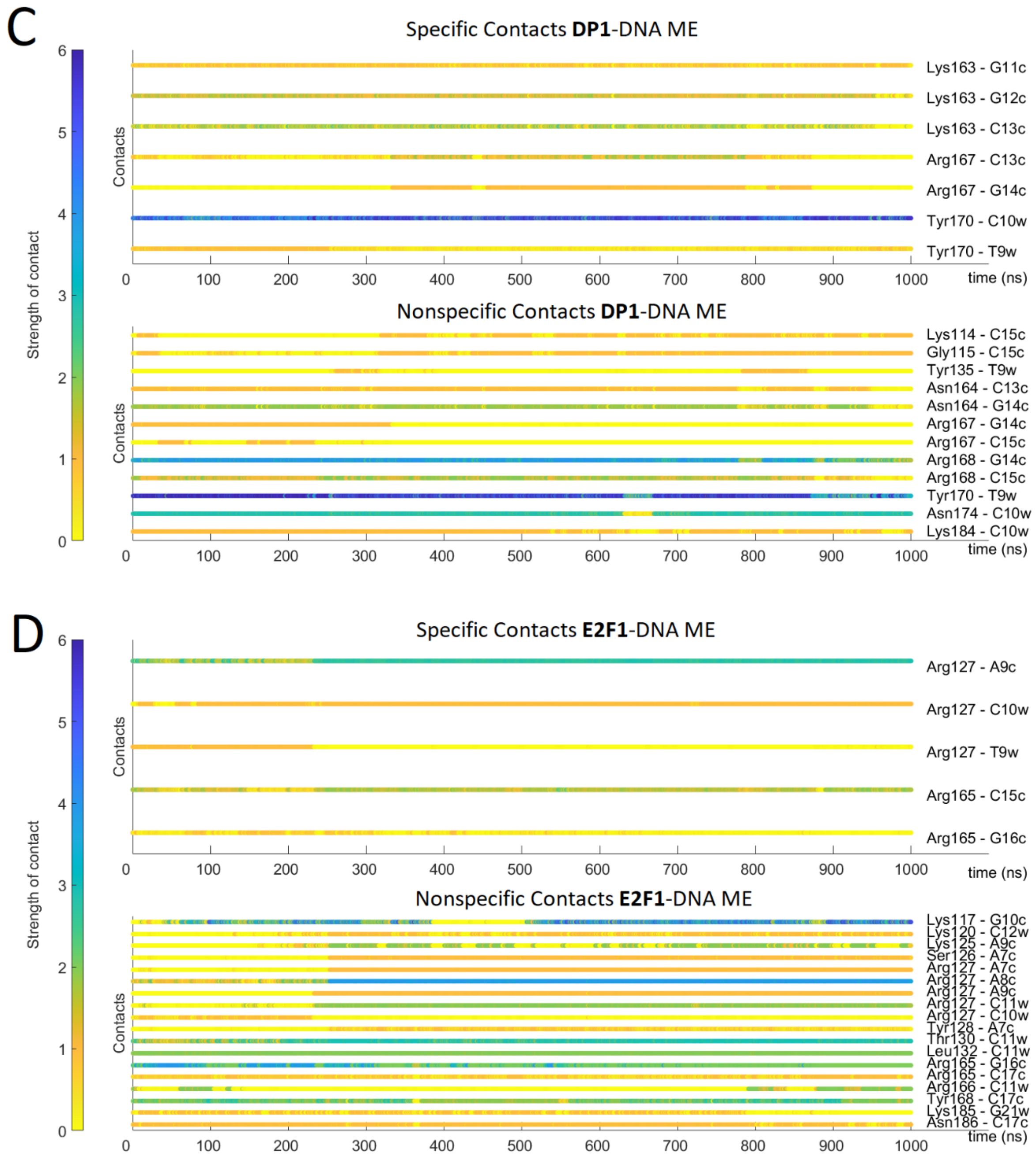

Figure S9. Time-evolution of the specific and nonspecific protein-DNA contacts for the E2F1-DP1-DNA system: **A:** WT DP1-DNA; **B:** WT E2F1-DNA; **C:** ME DP1-DNA; **D.** ME E2F1-DNA.

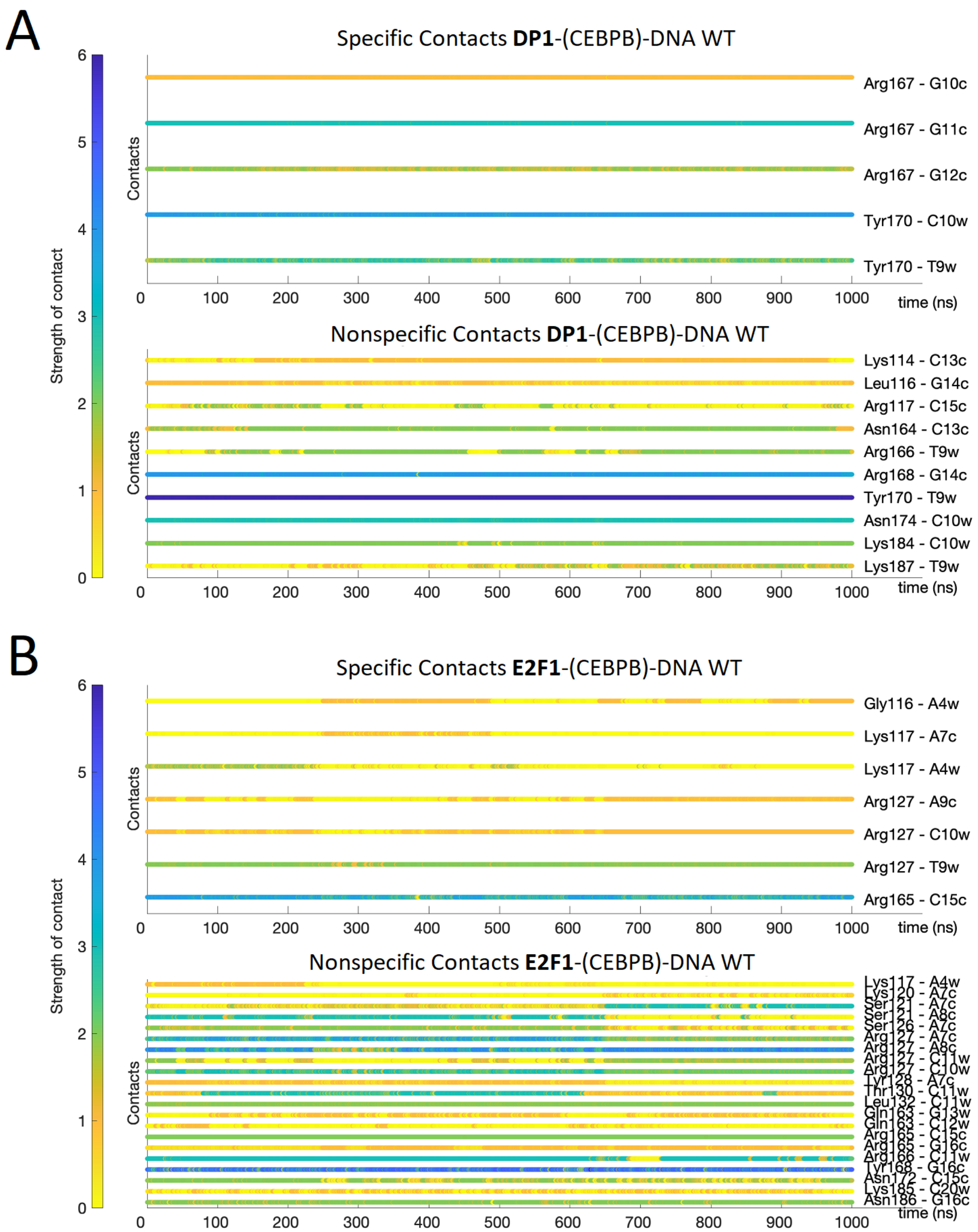
A
Specific Contacts DP1-(CEBPB)-DNA WT
Arg167 - G10c
Arg167 - G11c
Arg167 - G12c
Tyr170 - C10w
Tyr170 - T9w
Nonspecific Contacts DP1-(CEBPB)-DNA WT
Lys114 - C13c
Leu116 - G14c
Arg117 - C15c
Asn164 - C13c
Arg166 - T9w
Arg168 - G14c
Tyr170 - T9w
Asn174 - C10w
Lys184 - C10w
Lys187 - T9w
Strength of contact
Contacts
time (ns)
B
Specific Contacts E2F1-(CEBPB)-DNA WT
Gly116 - A4w
Lys117 - A7c
Lys117 - A4w
Arg127 - A9c
Arg127 - C10w
Arg127 - T9w
Arg165 - C15c
Nonspecific Contacts E2F1-(CEBPB)-DNA WT
Lys117 - A4w
Lys120 - A7c
Ser121 - A7c
Ser121 - A8c
Ser126 - A7c
Arg127 - A7c
Arg127 - A8c
Arg127 - C11w
Arg127 - C10w
Tyr128 - A7c
Thr130 - C11w
Leu132 - C11w
Gln163 - G13w
Gln163 - C12w
Arg165 - C15c
Arg165 - G16c
Arg166 - C11w
Tyr168 - G16c
Asn172 - C15c
Lys185 - C20w
Asn186 - G16c
Strength of contact
Contacts
time (ns)

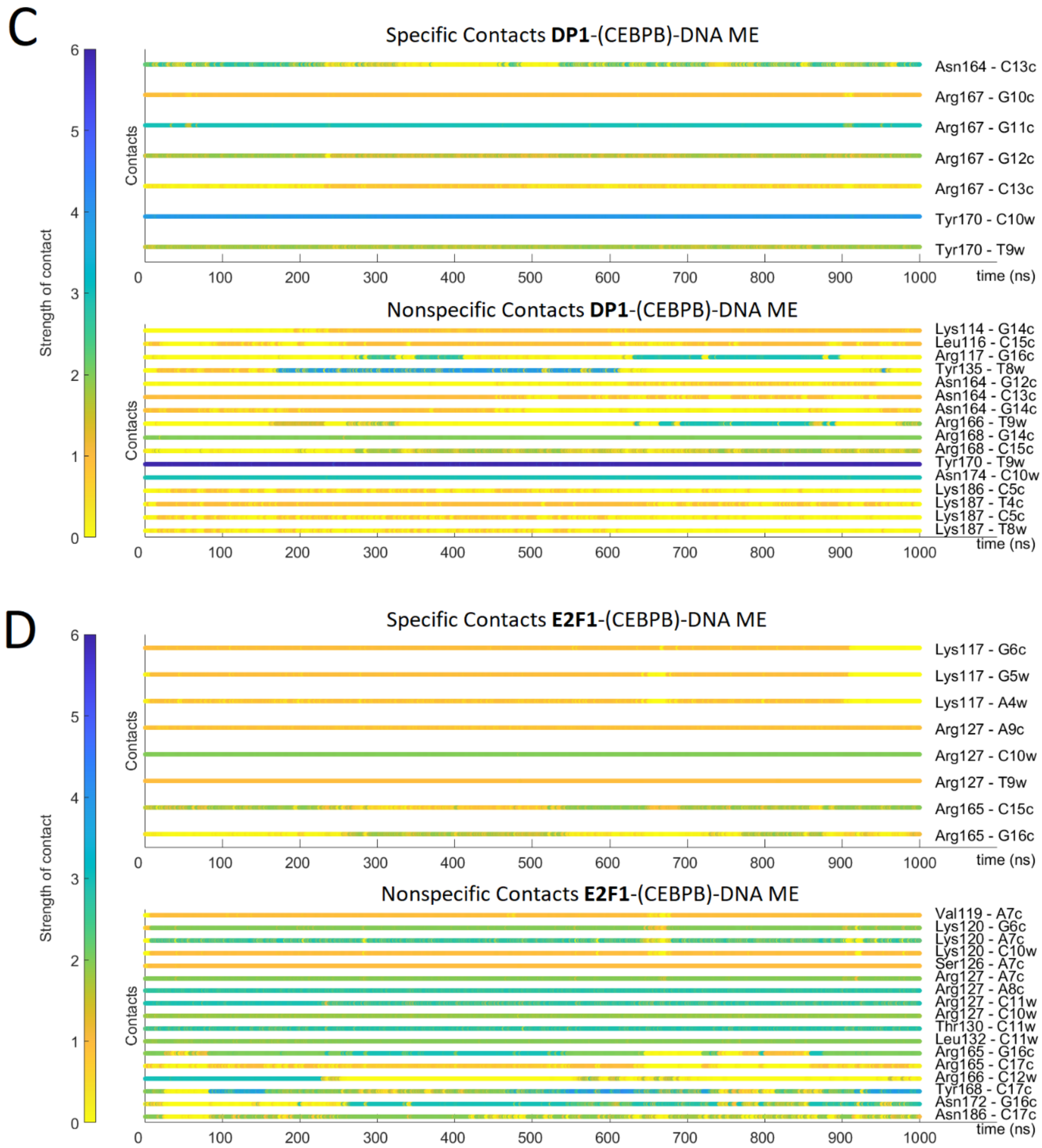

Figure S10. Time-evolution of the specific and nonspecific protein-DNA contacts for the E2F1-DP1-CEBPB-DNA system: **A:** WT DP1-DNA; **B:** WT E2F1-DNA; **C:** ME DP1-DNA; **D:** ME E2F1-DNA.

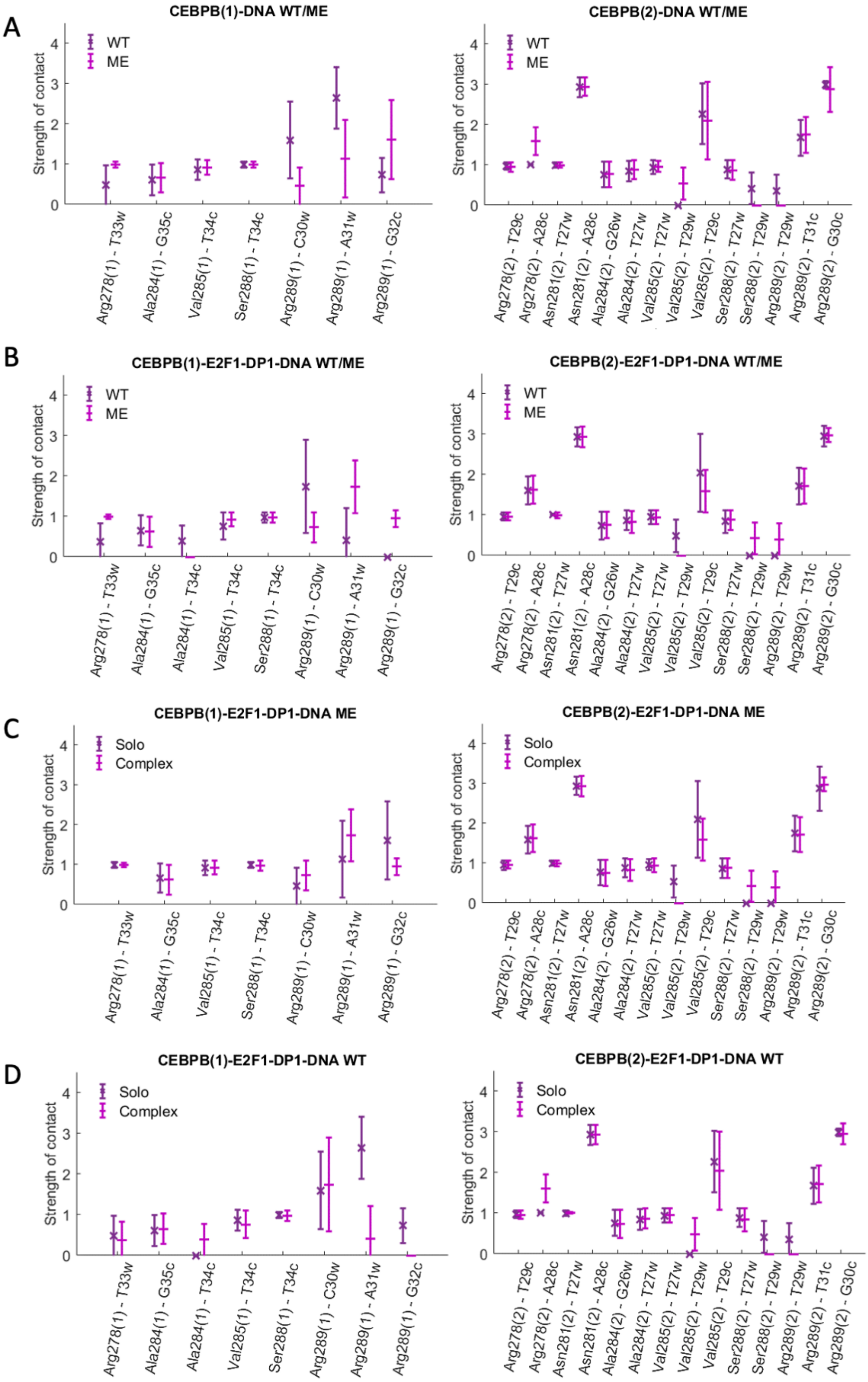

Figure S11. Specific contacts between CEBPB-dimer and DNA. The plots show the strength of specific contacts formed by the two CEBPB monomers, marked with (1) and (2), in wild-type (WT) and methylated (ME) cases, when bound alone to DNA (solo) and together with E2F1-DP1 (complex). For the definition of a contact strength see Supplementary Methods.

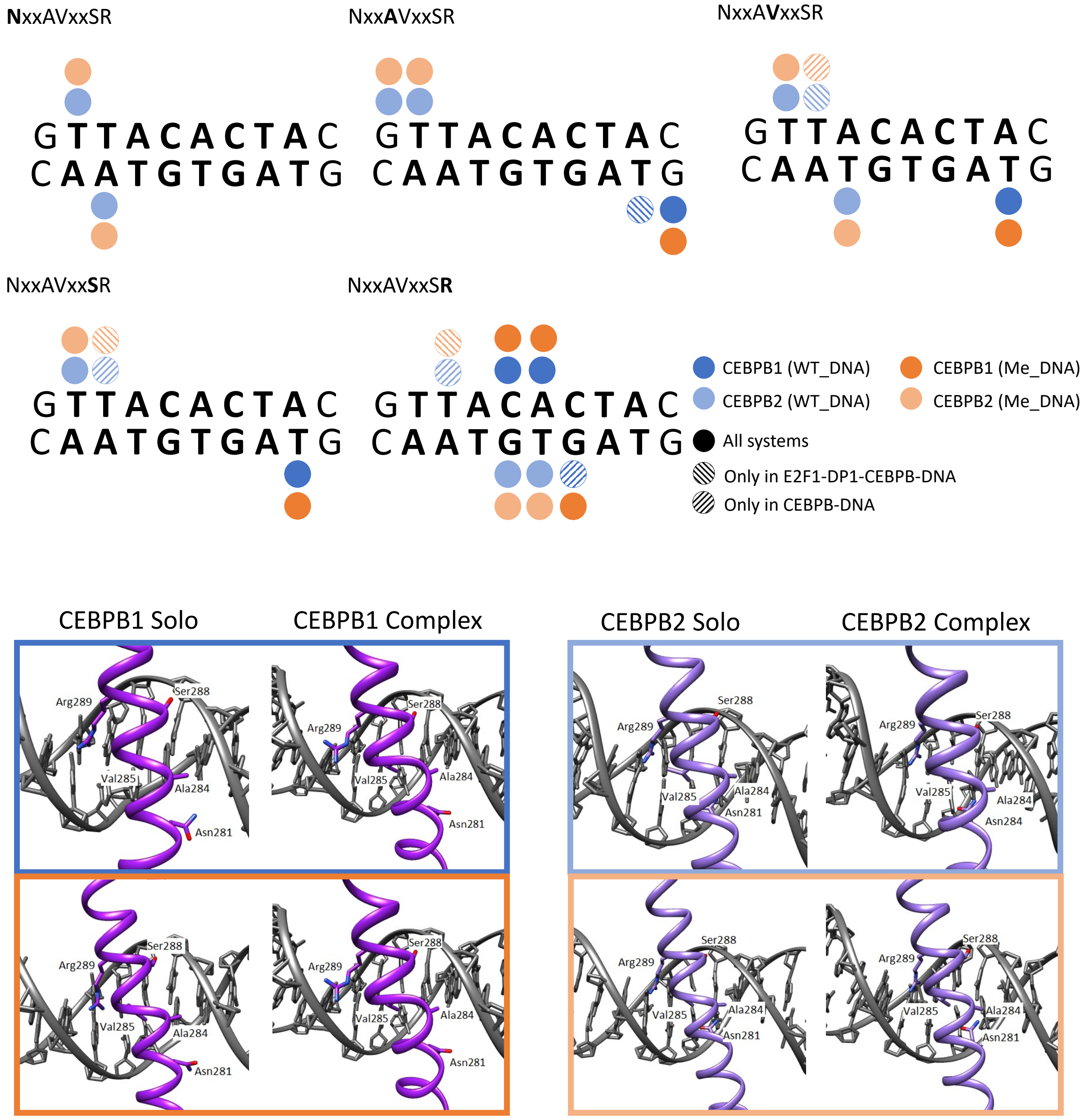

Figure S12: Above: Schematic representation of specific protein-DNA contacts exploited by the five-residues-motif (NxxAVxxSR) of CEBPB-dimer in different systems. **Below:** Cartoon representation of the binding orientations and DNA contacts of the CEBPB dimer for WT (blue) and ME (orange) systems, when bound alone to DNA (solo) and together with E2F1-DP1 (complex).

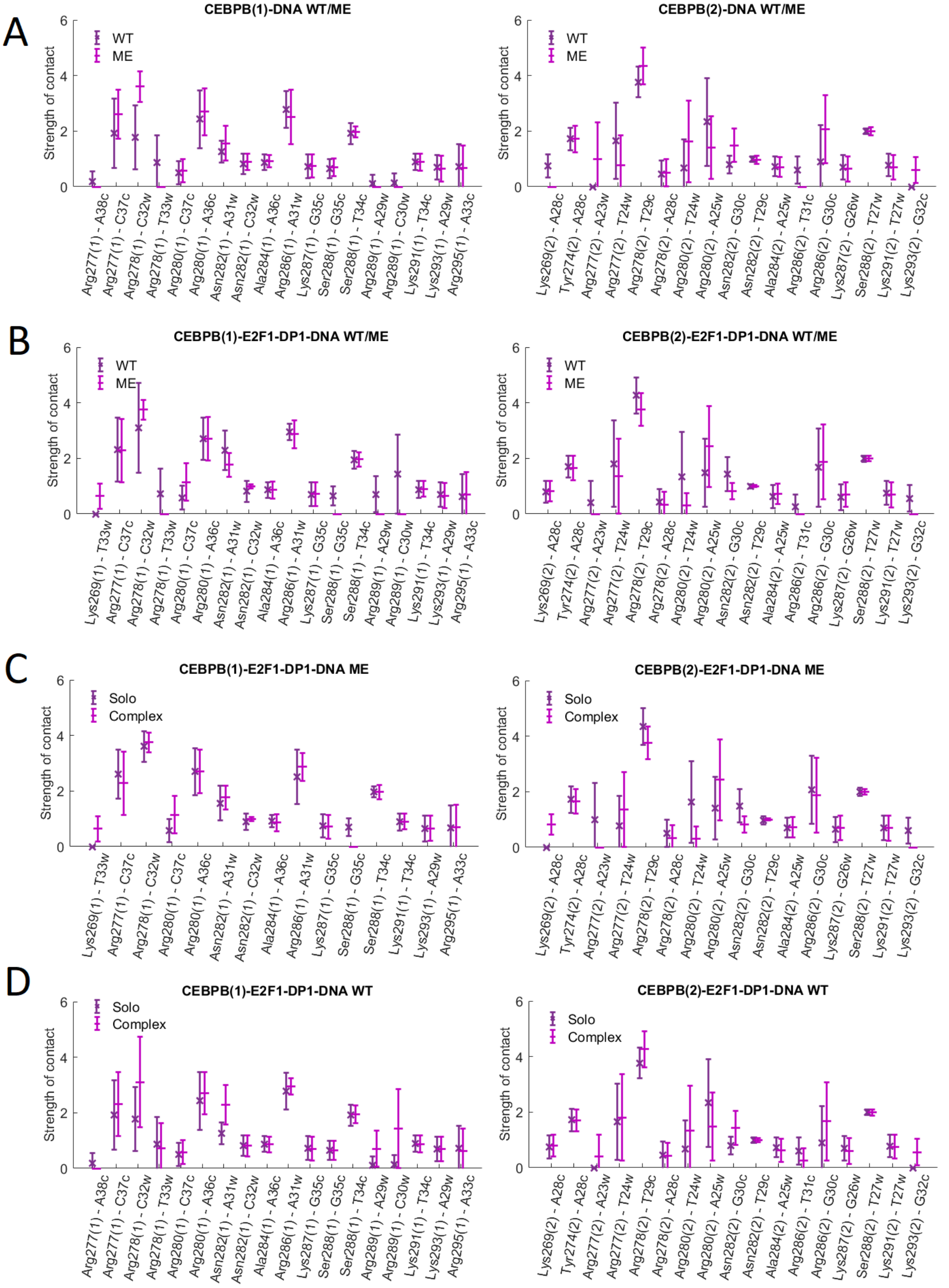

Figure S13: Nonspecific contacts between CEBPB-dimer and DNA. The plots show the strength of nonspecific contacts formed by the two CEBPB monomers, marked with (1) and (2), in wild-type (WT) and methylated (ME) cases, when bound alone to DNA (solo) and together with E2F1-DP1 (complex). For the definition of a contact strength see Supplementary Methods.

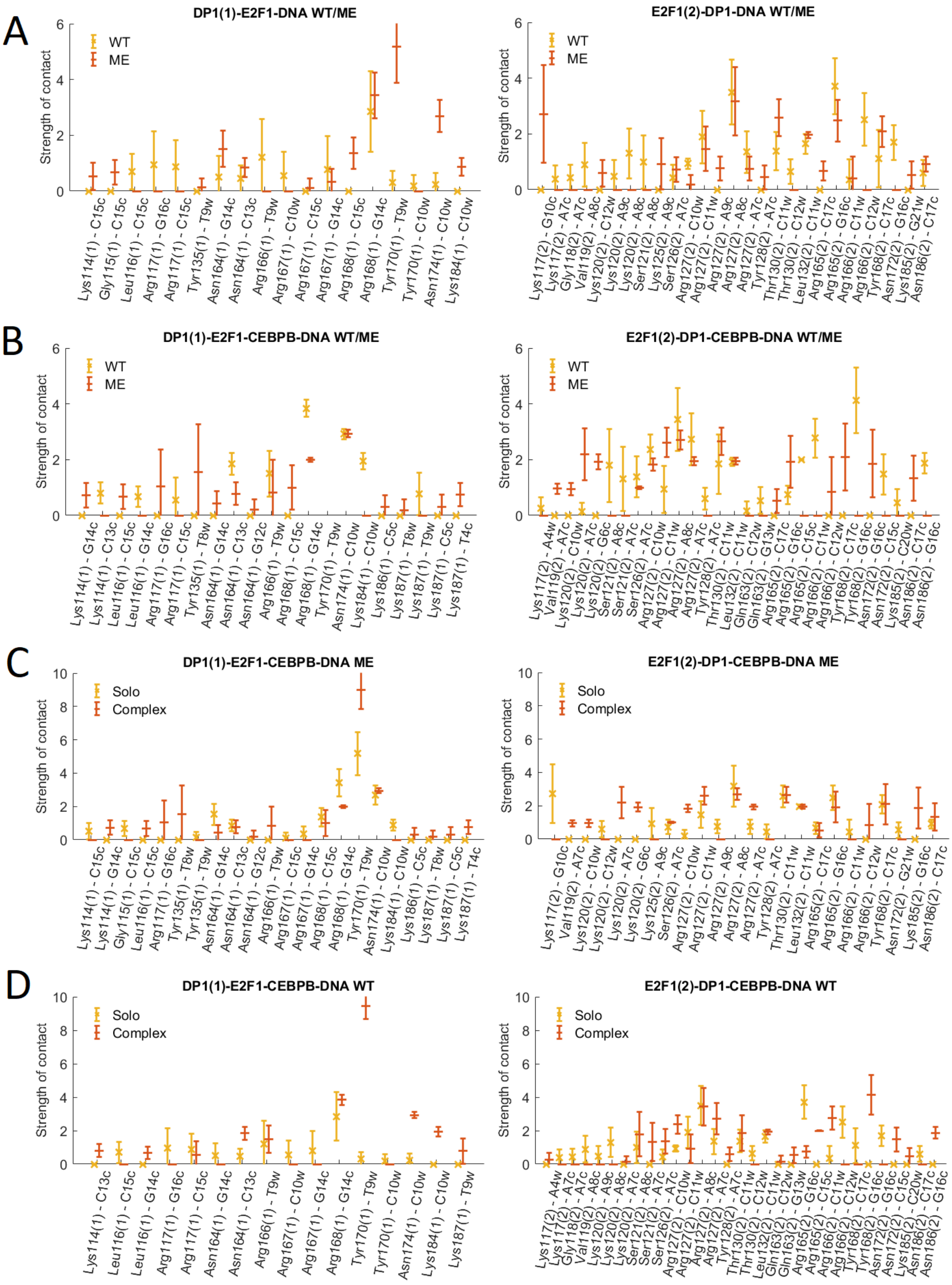

Figure S14: Nonspecific contacts between E2F1-DP1-dimer and DNA. The plots show the strength of nonspecific contacts formed by the E2F1 and DP1 monomers, marked with (1) and (2), in wild-type (WT) and methylated (ME) cases, when bound alone to DNA (solo) and together with E2F1-DP1 (complex). For the definition of a contact strength see Supplementary Methods.

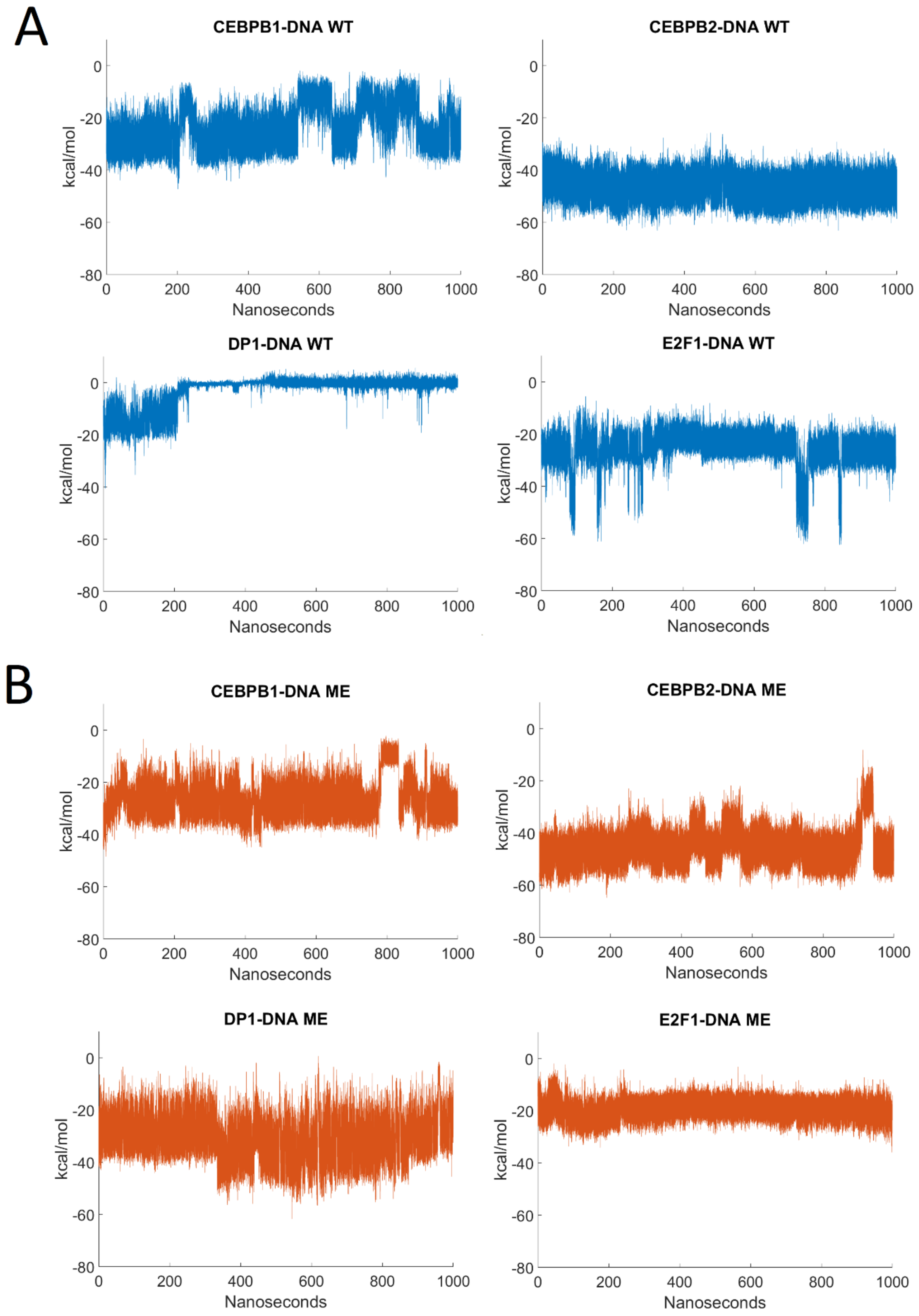

Figure S15: Time-evolution of specific interaction energies for the different solo bound TF-DNA complexes for **A:** WT systems (blue) and **B:** ME systems (red).

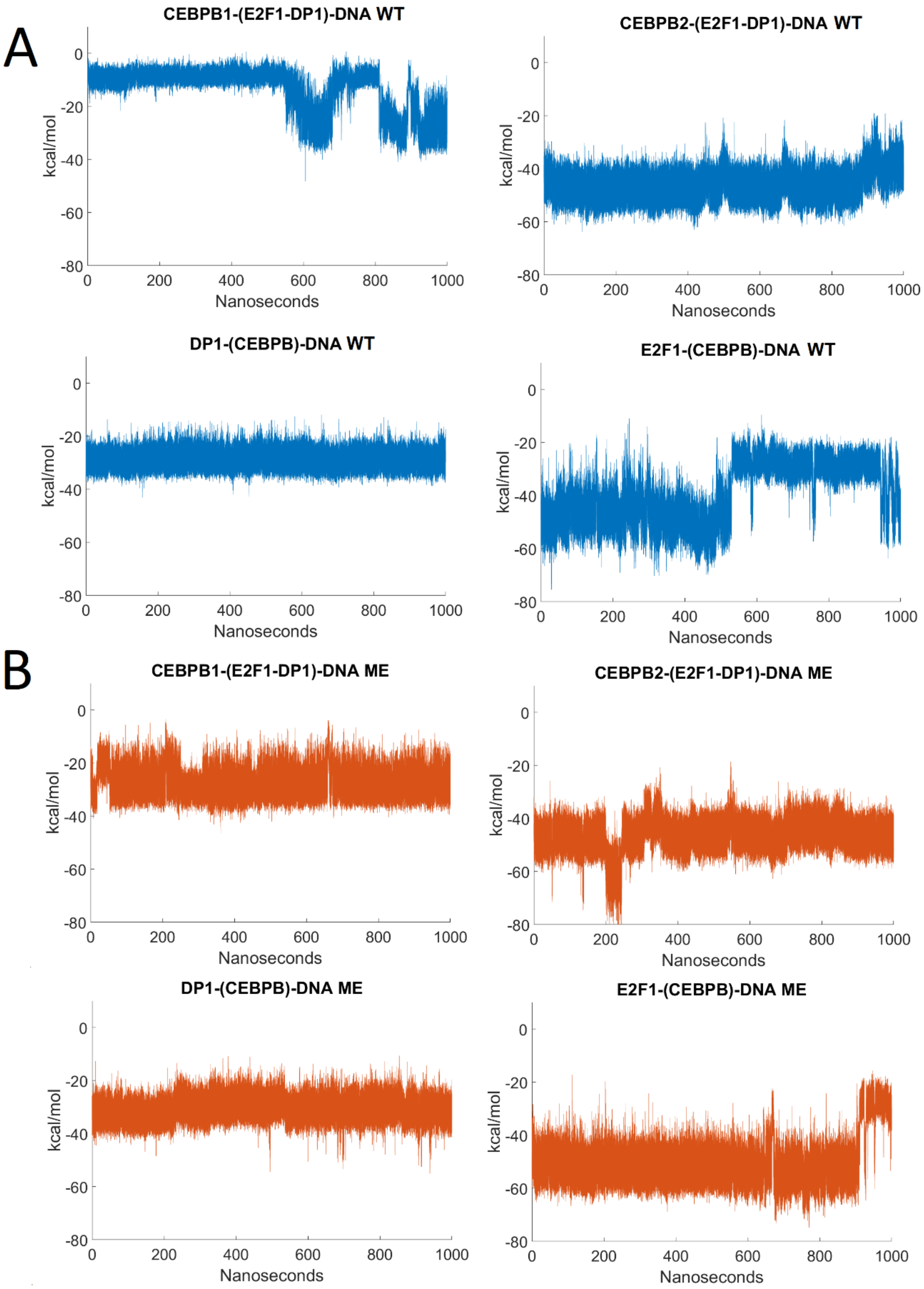

Figure S16: Time-evolution of specific interaction energies for the different TF-DNA complexes indicated in parentheses for complete enhanceosome complexes (CEBPB-E2F1-DP1-DNA) for **A:** WT systems (blue) and **B:** ME systems (red).

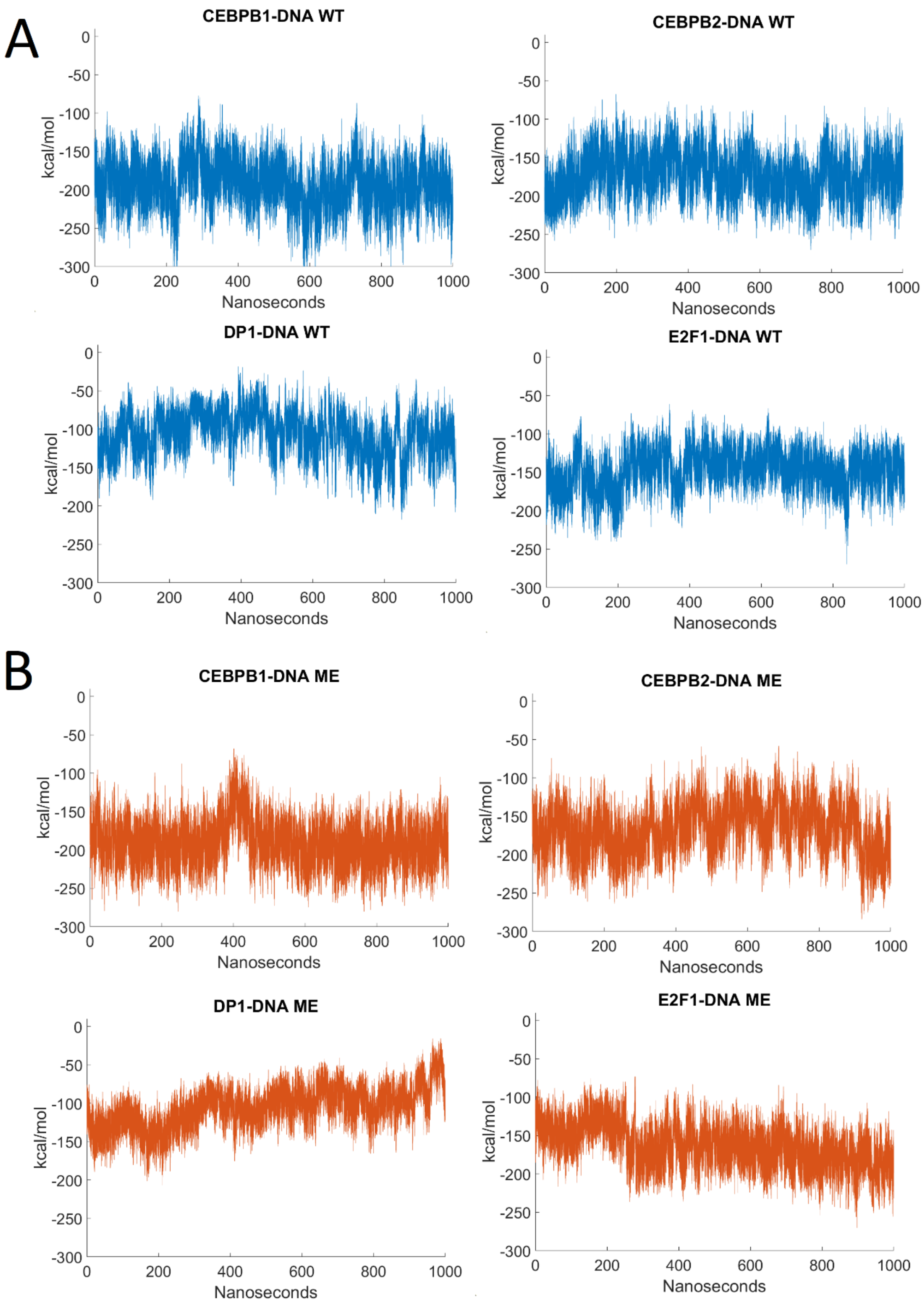

Figure S17: Time-evolution of nonspecific interaction energies for the different solo bound TF-DNA complexes for **A:** WT systems (blue) and **B:** ME systems (red).

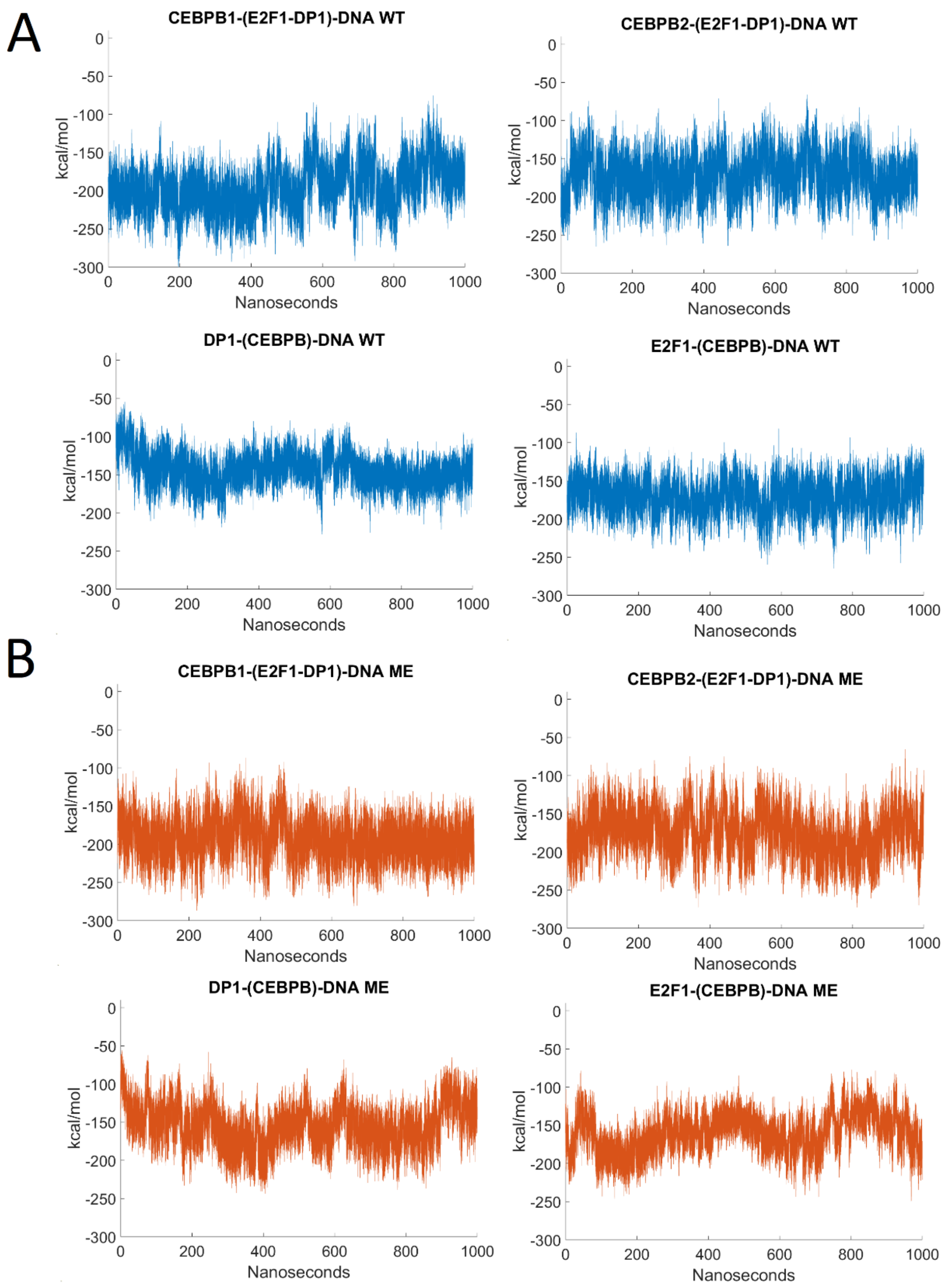

Figure S18: Time-evolution of nonspecific interaction energies for the different TF-DNA complexes indicated in parentheses for complete enhanceosome complexes (CEBPB-E2F1-DP1-DNA) for **A:** WT systems (blue) and **B:** ME systems (red).

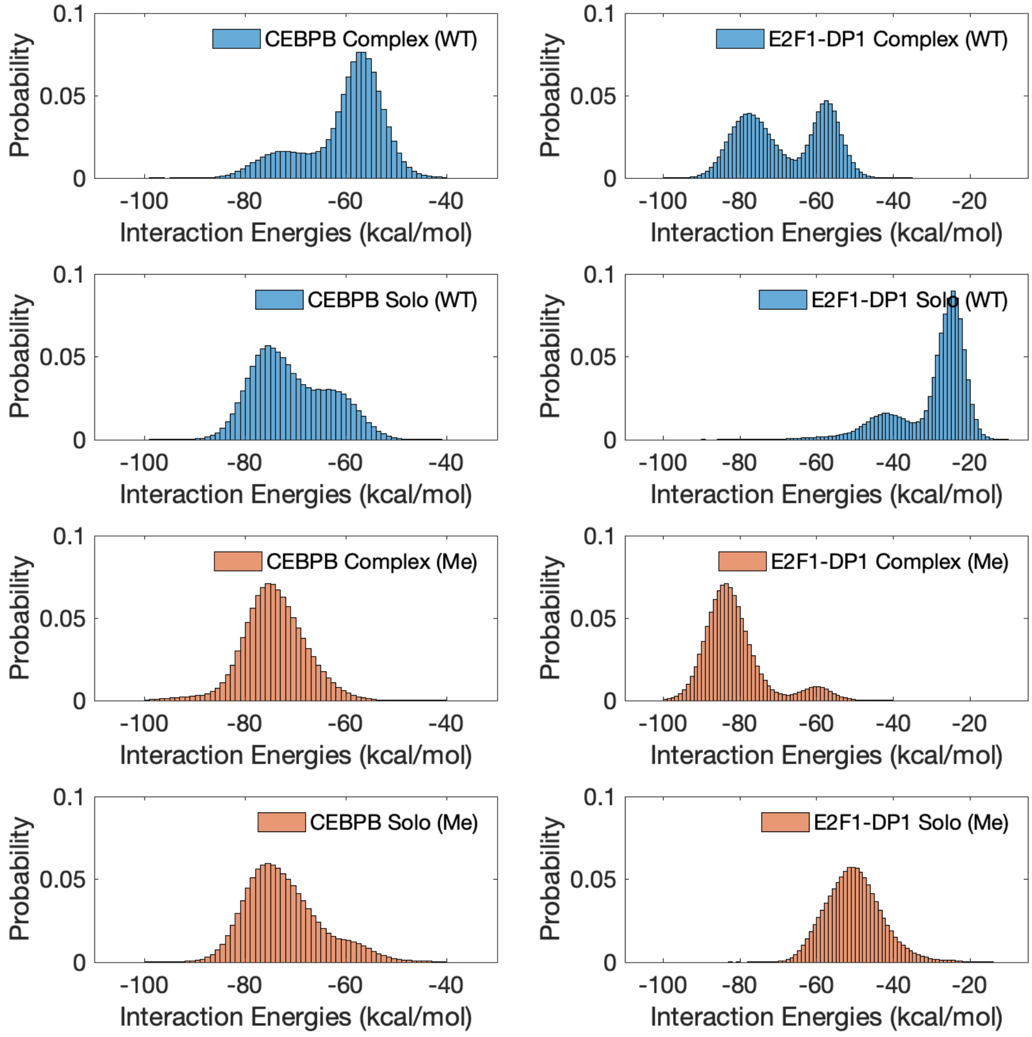

Figure S19: Specific contacts interaction energies distributions for the corresponding TF-dimer-DNA complex, were "Solo" corresponds to either CEBPB-DNA or E2F1-DP1-DNA systems, and "Complex" – to CEBPB-E2F1-DP1-DNA systems, the "WT" and "ME" denote wild type and methylated DNA systems.

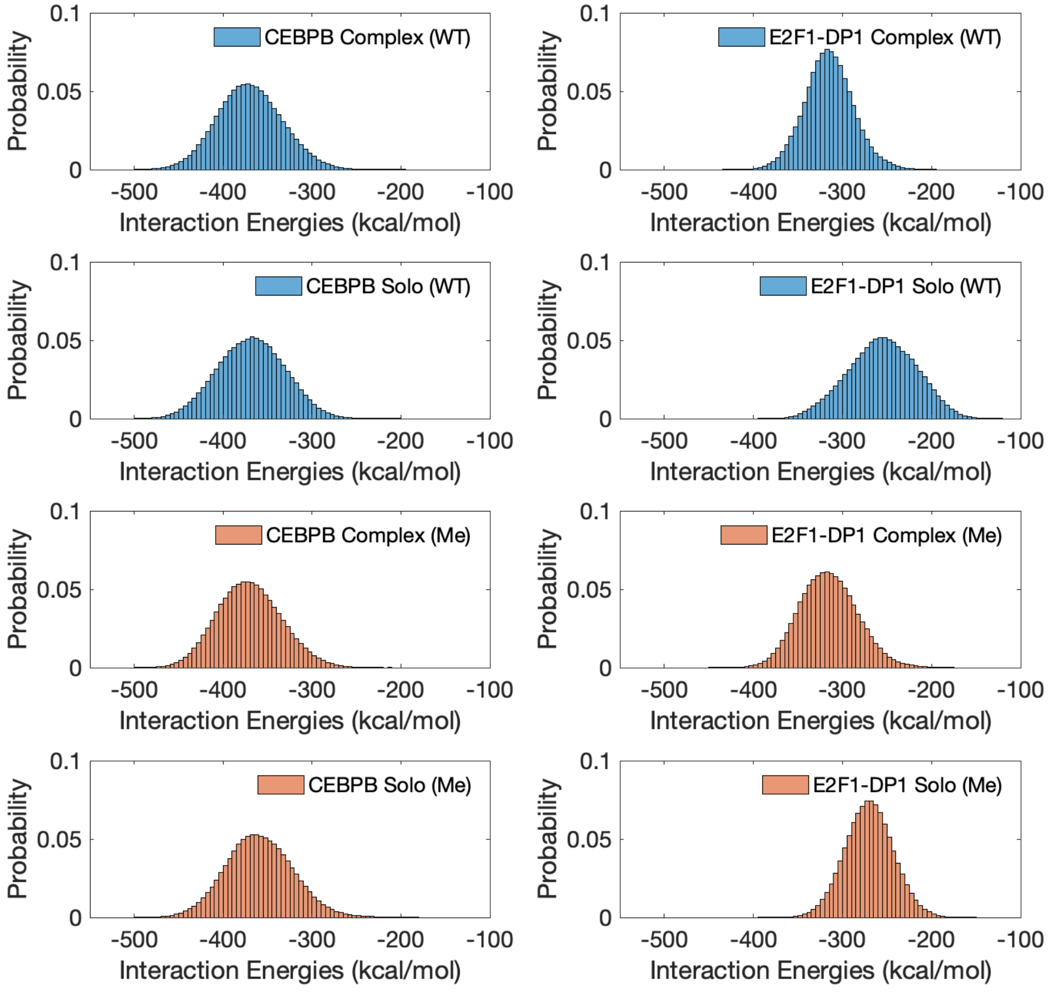

Figure S20: Nonspecific contacts interaction energies distributions for the corresponding TF-dimer-DNA complex, were "Solo" corresponds to either CEBPB-DNA or E2F1-DP1-DNA systems, and "Complex" – to CEBPB-E2F1-DP1-DNA systems, the "WT" and "ME" denote wild type and methylated DNA systems.

Table S2: Protein-DNA binding energies (kcal/mol) calculated according the MM(GB/PB)SA approach for wild-type (WT) and methylated (ME) systems. For E2F1-DP1-CEBPB-DNA trajectories, the provided interaction energies are for the protein dimer in bold.

	**CEBPB-DNA**	**E2F1-DP1-DNA**	E2F1-DP1-**CEBPB-DNA**	CEBPB-**E2F1-DP1-DNA**
			**MMGBSA**	
**WT**	-276.5±14.8	-181.1±15.1	-276.0±16.1	-215.4±12.6
**ME**	-277.6±15.9	-194.6±15.5	-281.4±13.3	-214.2±17.0
			**MMPBSA**	
**WT**	-285.1±15.7	-204.2±16.1	-281.5±15.0	-225.4±14.1
**ME**	-285.5±16.1	-204.4±16.2	-289.7±14.4	-220.5±17.8

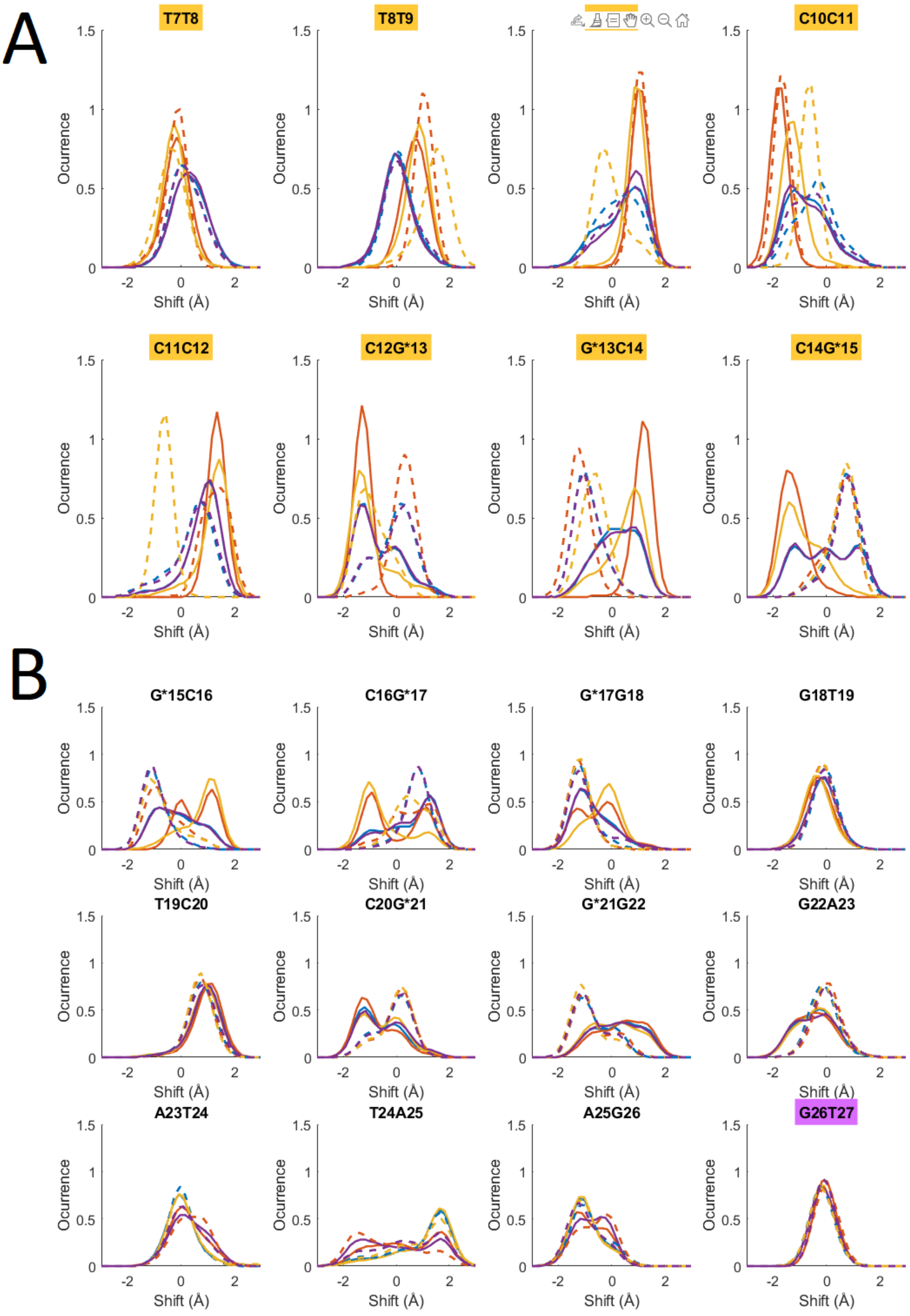
A
T7T8
T8T9
C10C11
C11C12
C12G*13
G*13C14
C14G*15
B
G*15C16
C16G*17
G*17G18
G18T19
T19C20
C20G*21
G*21G22
G22A23
A23T24
T24A25
A25G26
G26T27
Ocurrence
Shift (Å)

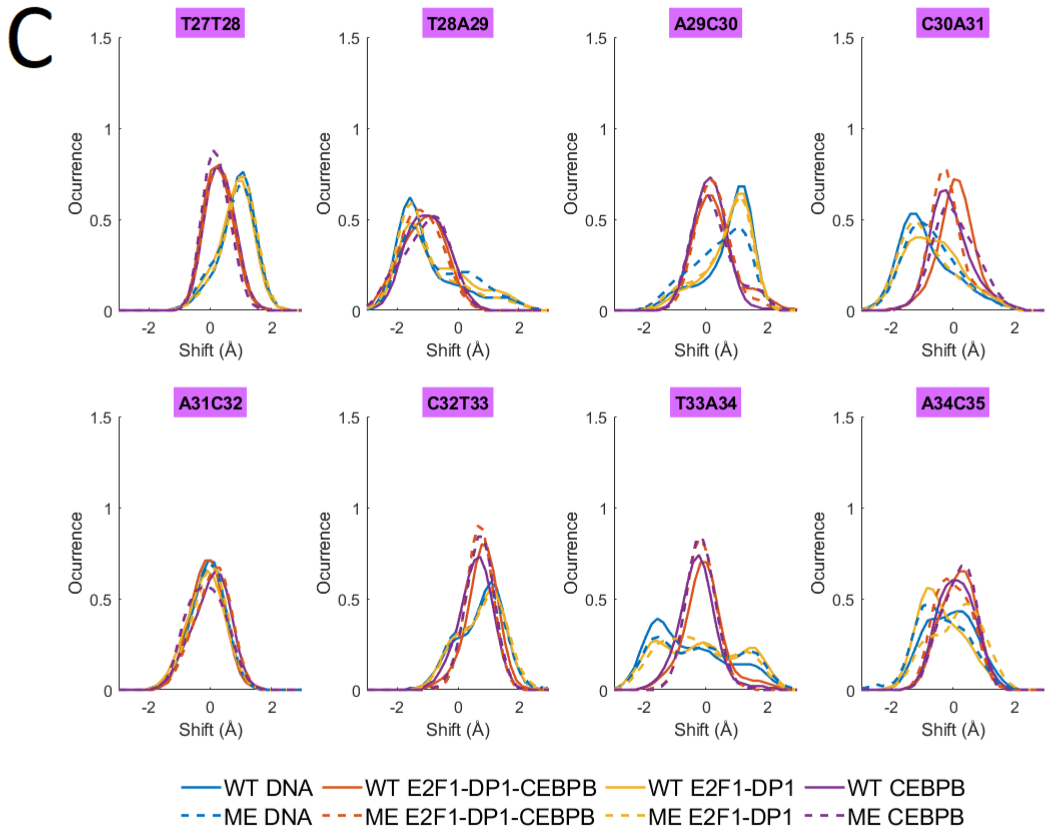

Figure S21: Normalized b.p. step shift distributions for DNA alone and in complex with either one (CEBPB or E2F1-DP1 TFs dimer) or both for **A:** the E2F1-DP1 binding site; **B:** the linker region; **C:** the CEBPB binding site.

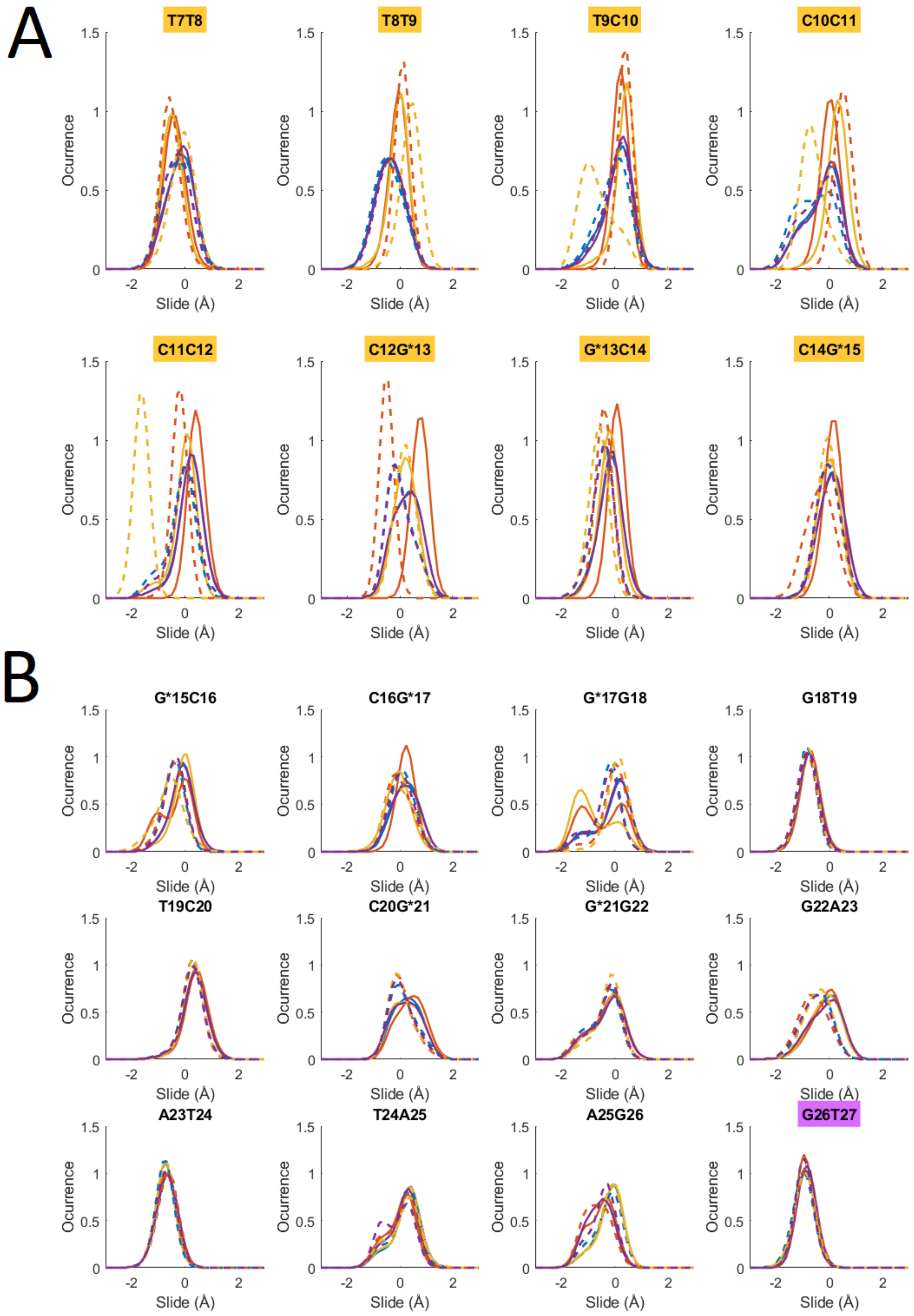

A
T7T8
T8T9
T9C10
C10C11
C11C12
C12G*13
G*13C14
C14G*15
B
G*15C16
C16G*17
G*17G18
G18T19
T19C20
C20G*21
G*21G22
G22A23
A23T24
T24A25
A25G26
G26T27
Ocurrence
Slide (Å)

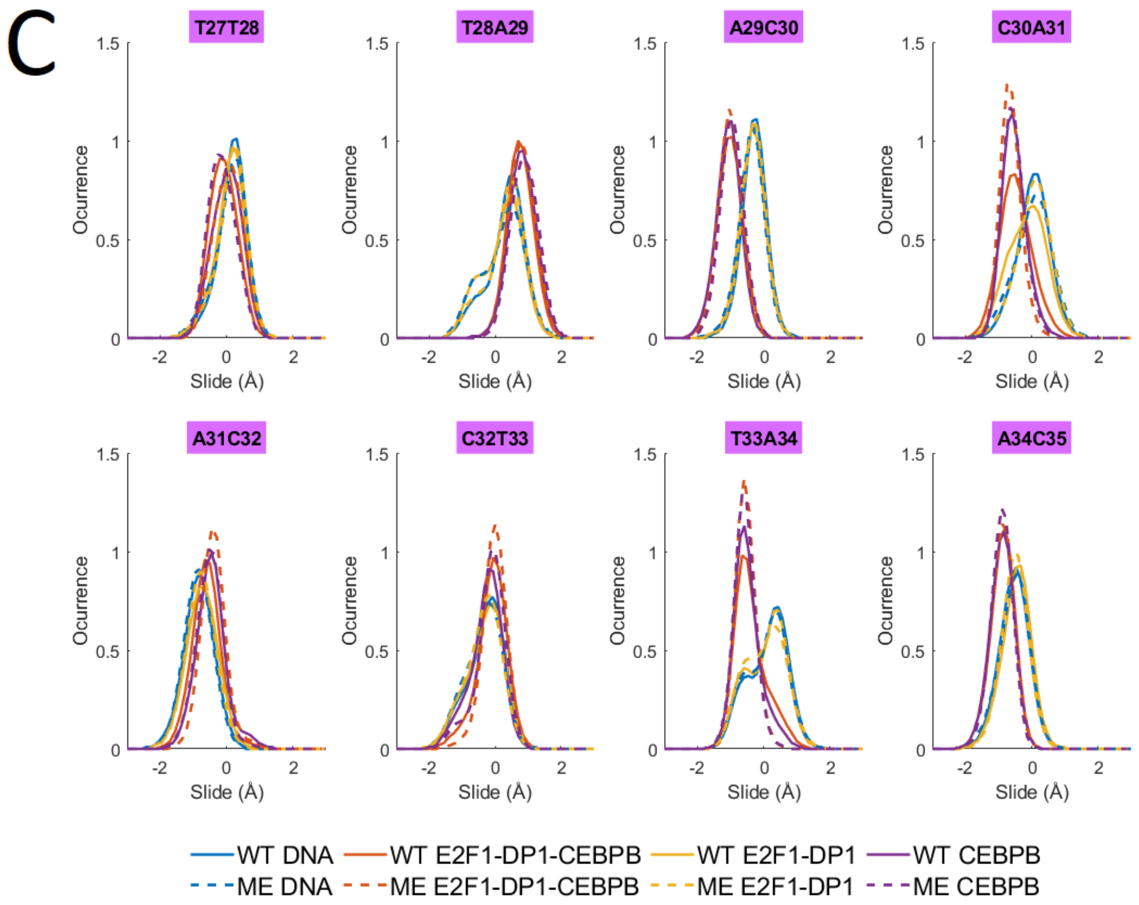

Figure S22: Normalized b.p. step slide distributions for DNA alone and in complex with either one (CEBPB or E2F1-DP1 TFs dimer) or both for **A:** the E2F1-DP1 binding site; **B:** the linker region; **C:** the CEBPB binding site.

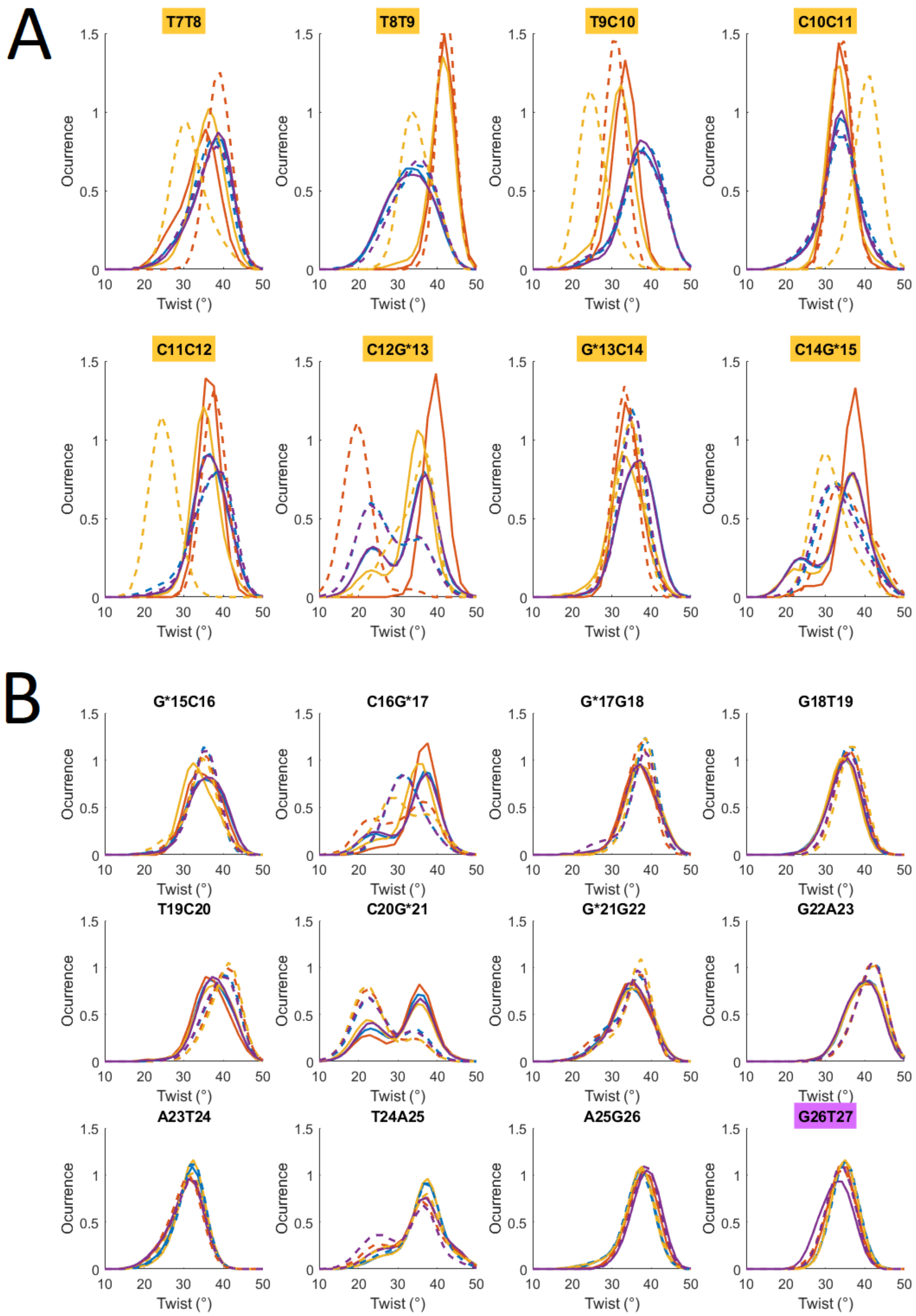
A
T7T8
T8T9
T9C10
C10C11
C11C12
C12G*13
G*13C14
C14G*15
B
G*15C16
C16G*17
G*17G18
G18T19
T19C20
C20G*21
G*21G22
G22A23
A23T24
T24A25
A25G26
G26T27
Ocurrence
Twist (°)

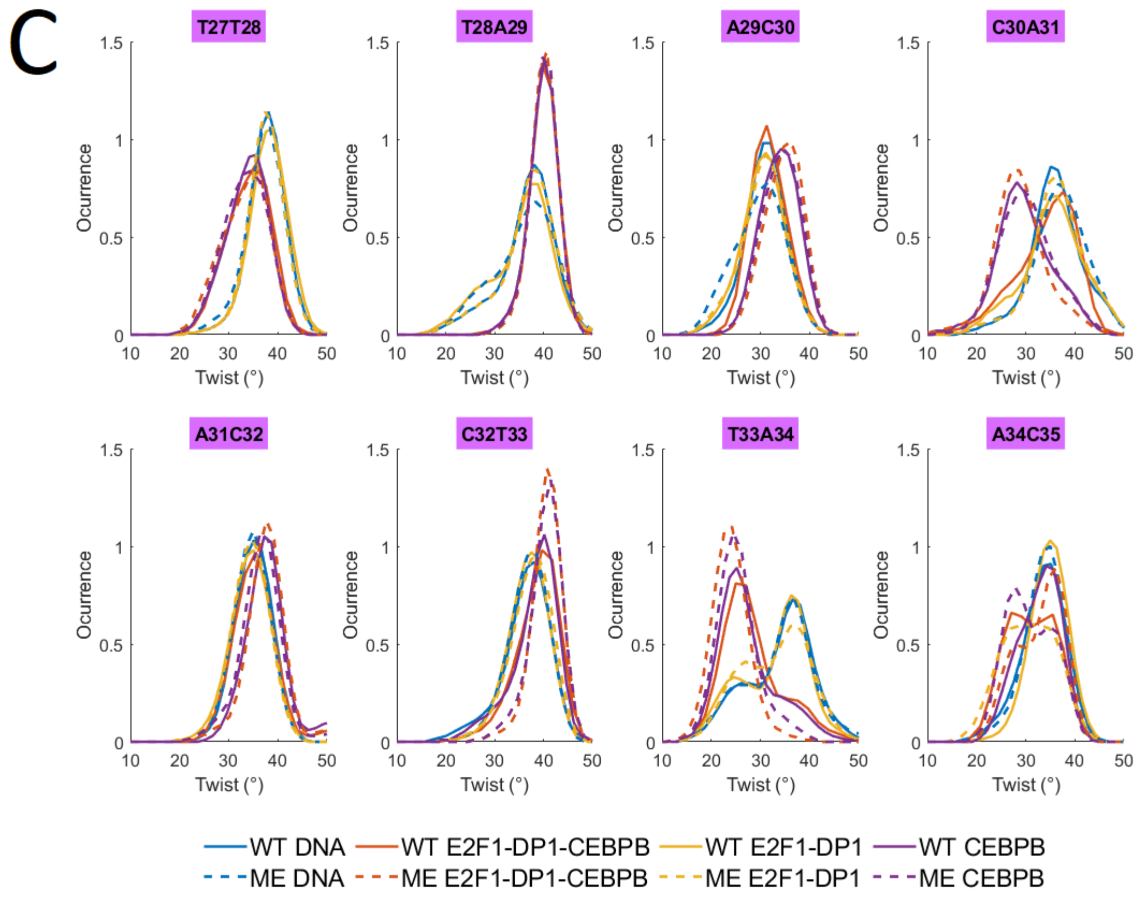

Figure S23: Normalized b.p. step twist distributions for DNA alone and in complex with either one (CEBPB or E2F1-DP1 TFs dimer) or both for **A:** the E2F1-DP1 binding site; **B:** the linker region; **C:** the CEBPB binding site.

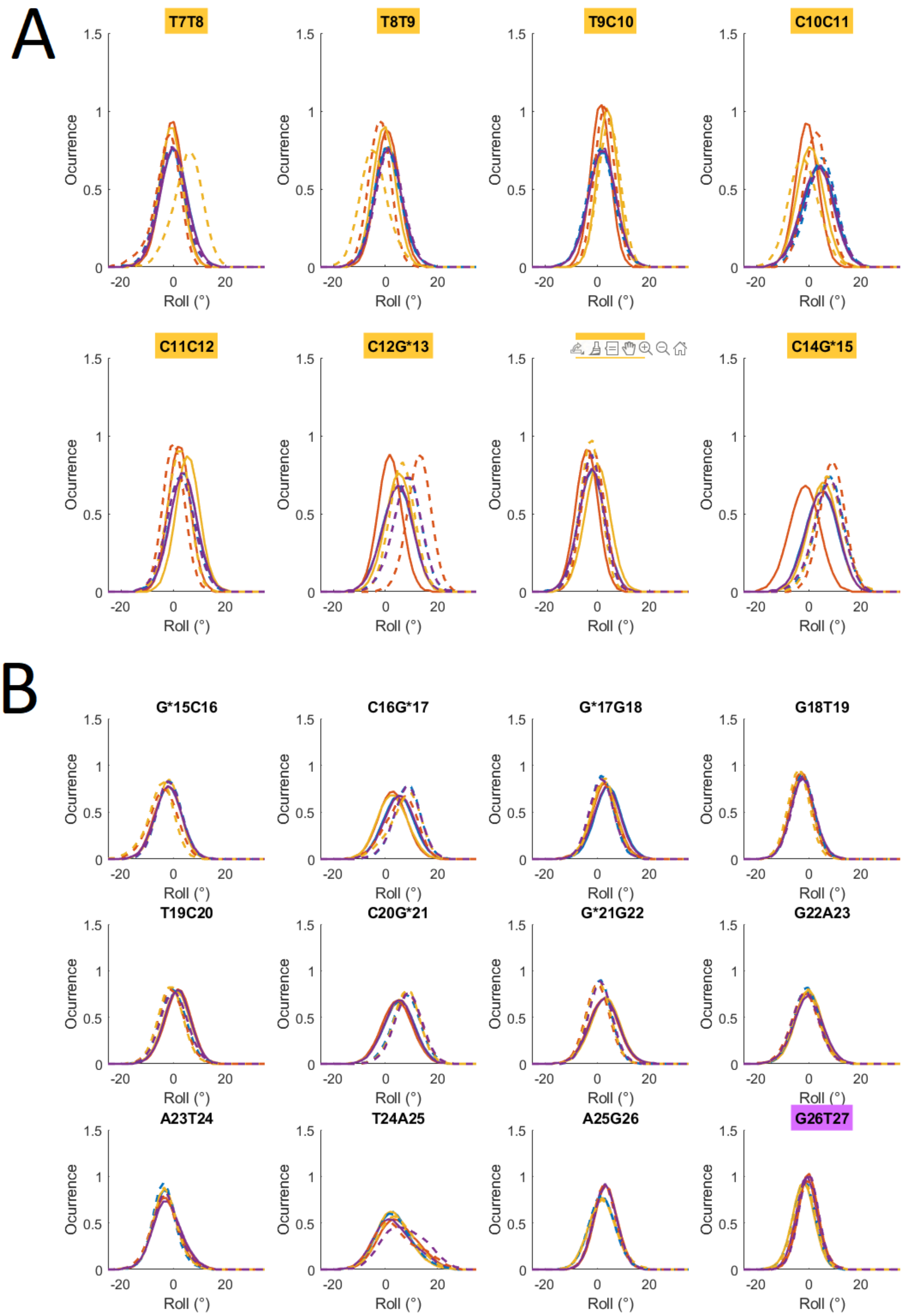
A
T7T8
T8T9
T9C10
C10C11
C11C12
C12G*13
C14G*15
B
G*15C16
C16G*17
G*17G18
G18T19
T19C20
C20G*21
G*21G22
G22A23
A23T24
T24A25
A25G26
G26T27
Ocurrence
Roll (°)

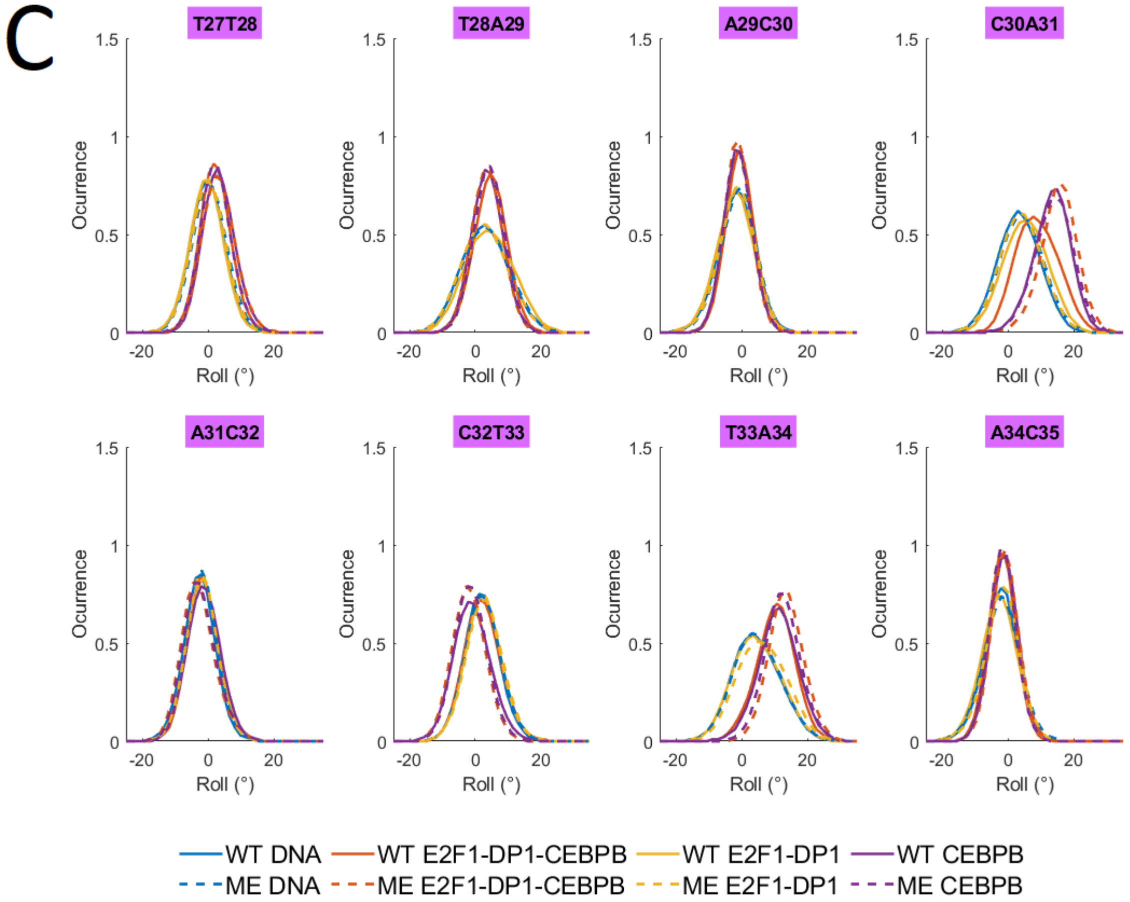

Figure S24: Normalized b.p. step roll distributions for DNA alone and in complex with either one (CEBPB or E2F1-DP1 TFs dimer) or both for **A:** the E2F1-DP1 binding site; **B:** the linker region; **C:** the CEBPB binding site.

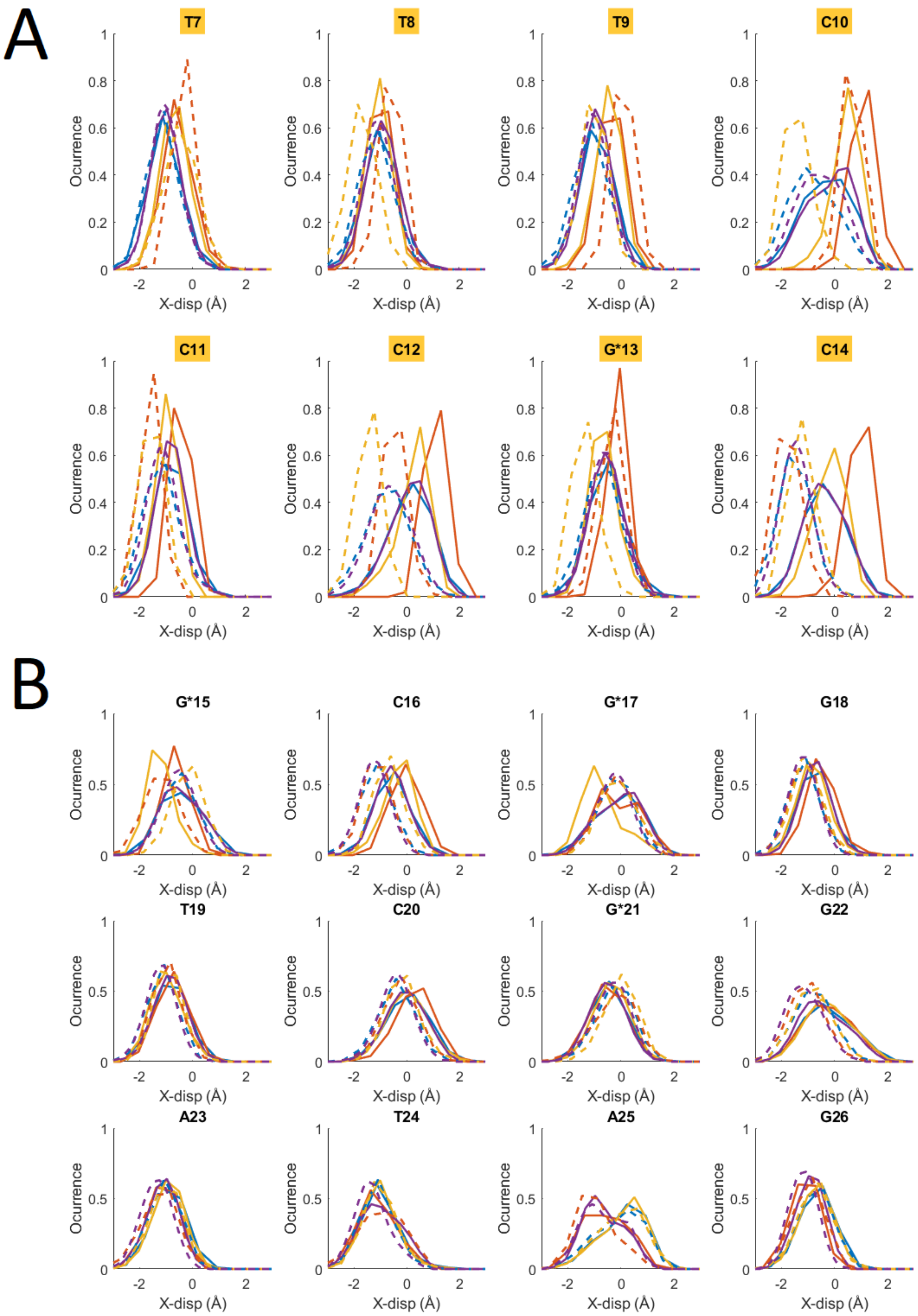
A
T7
T8
T9
C10
C11
C12
G*13
C14
B
G*15
C16
G*17
G18
T19
C20
G*21
G22
A23
T24
A25
G26
Ocurrence
X-disp (Å)

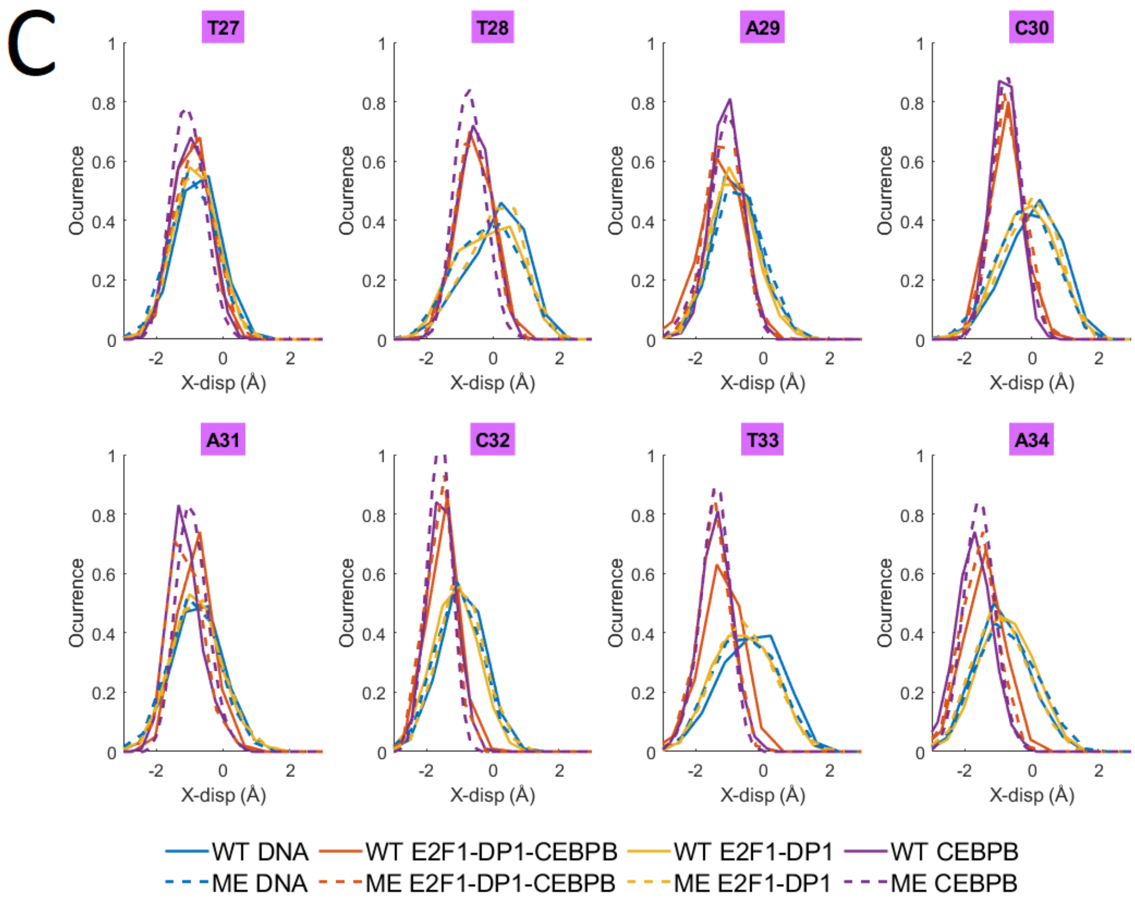

Figure S25: Normalized b.p. x-displacement distributions for DNA alone and in complex with either one (CEBPB or E2F1-DP1 TFs dimer) or both for **A:** the E2F1-DP1 binding site; **B:** the linker region; **C:** the CEBPB binding site.

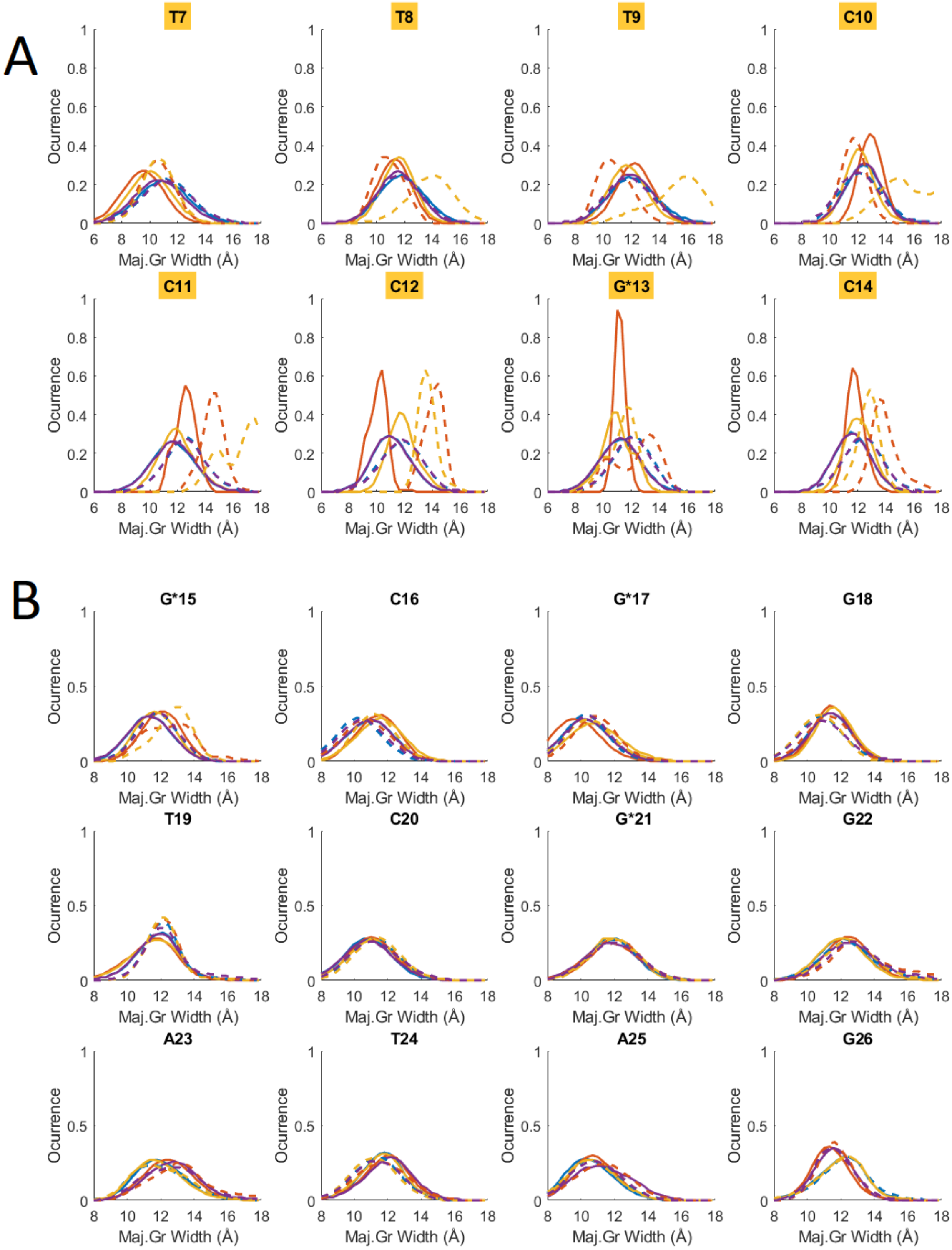
A
T7
T8
T9
C10
C11
C12
G*13
C14
B
G*15
C16
G*17
G18
T19
C20
G*21
G22
A23
T24
A25
G26
Ocurrence
Maj.Gr Width (Å)

C

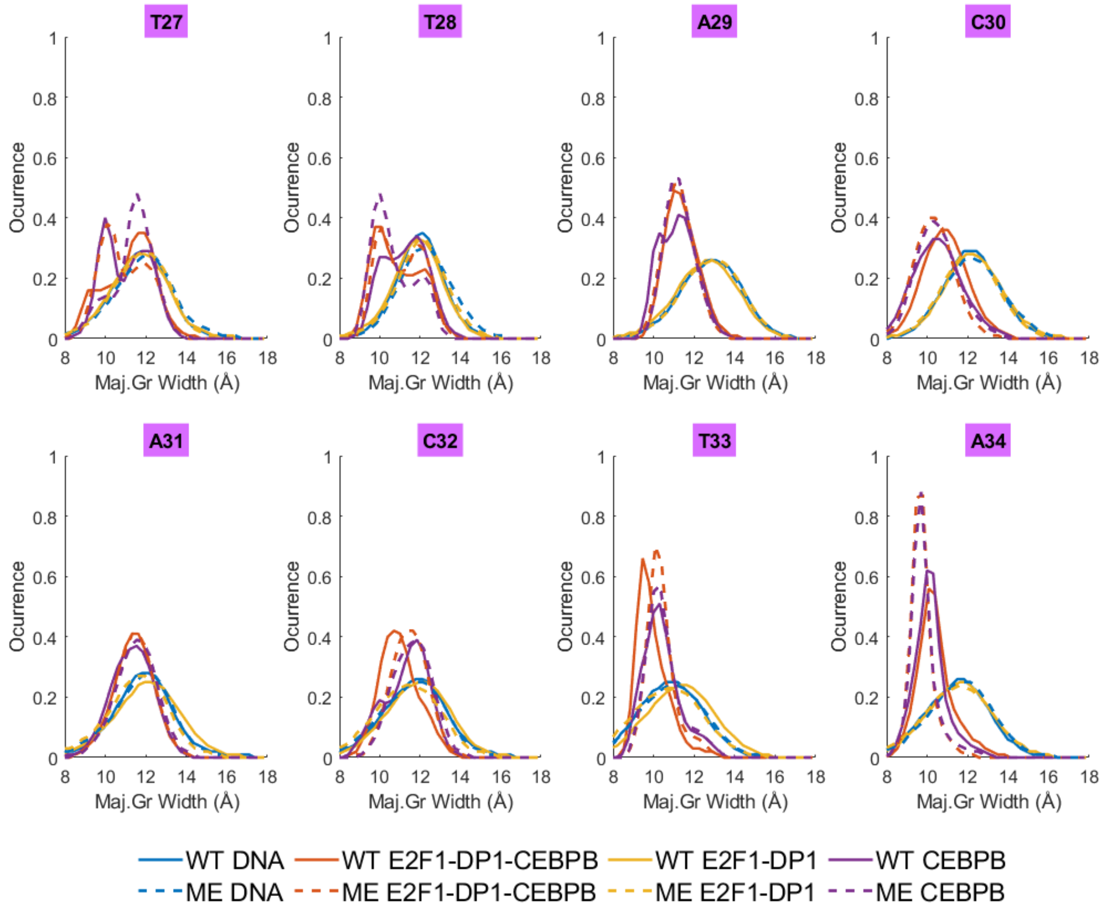

Figure S26: Normalized major groove width distributions for DNA alone and in complex with either one (CEBPB or E2F1-DP1 TFs dimer) or both for **A:** the E2F1-DP1 binding site; **B:** the linker region; **C:** the CEBPB binding site.

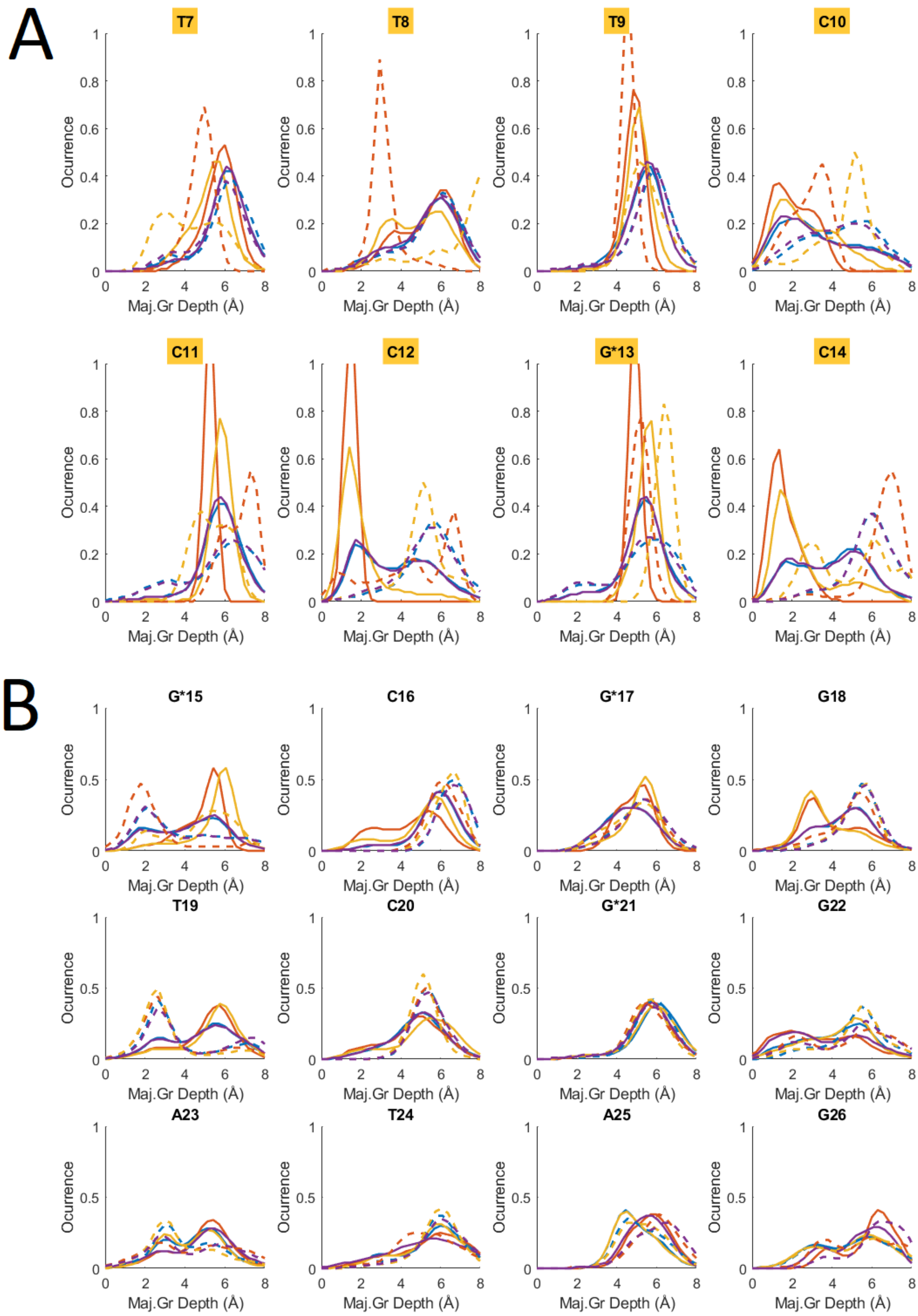
A
T7
T8
T9
C10
C11
C12
G*13
C14
B
G*15
C16
G*17
G18
T19
C20
G*21
G22
A23
T24
A25
G26
Ocurrence
Maj.Gr Depth (Å)
0
0.2
0.4
0.6
0.8
0.5
1
2
4
6
8

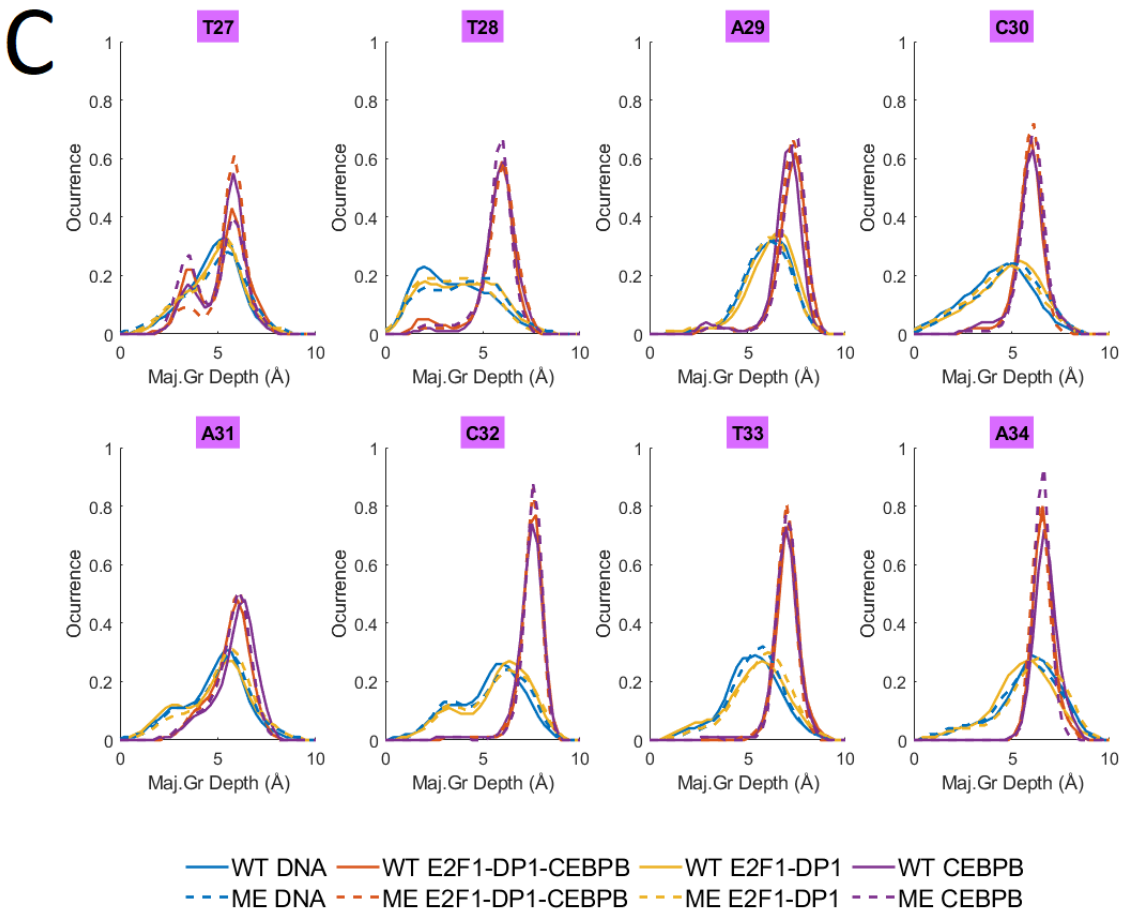

Figure S27: Normalized major groove depth distributions for DNA alone and in complex with either one (CEBPB or E2F1-DP1 TFs dimer) or both for **A:** the E2F1-DP1 binding site; **B:** the linker region; **C:** the CEBPB binding site.

Shift

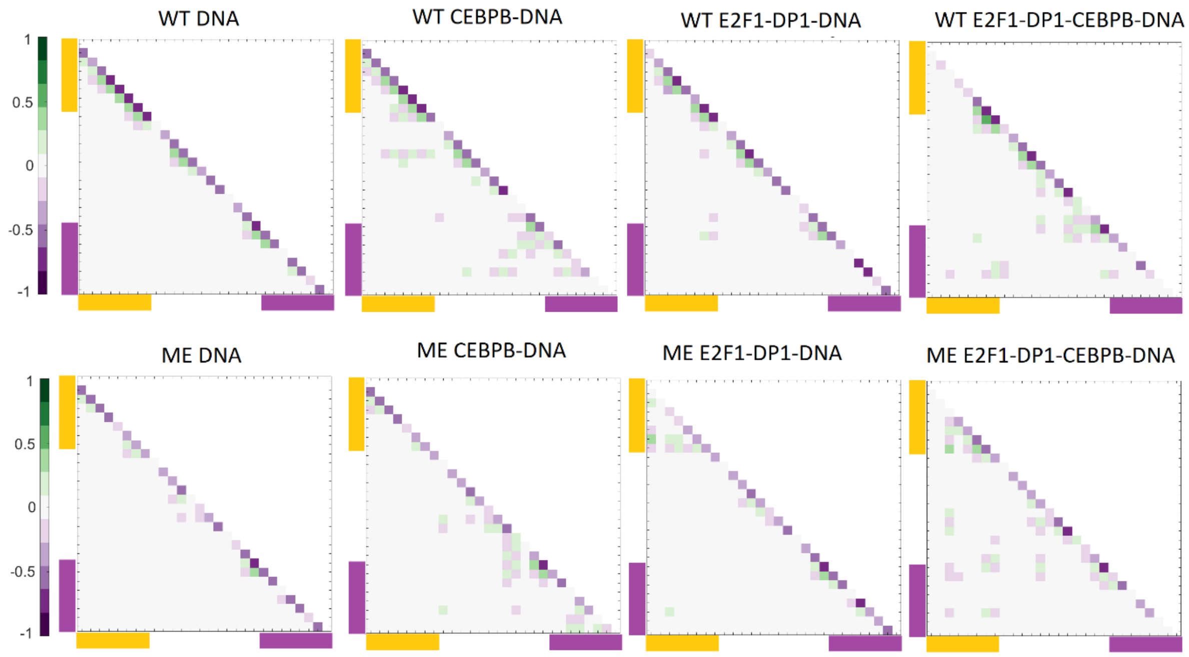

Figure S28: Time-delayed correlation coefficient maps for b.p. step shift, with a maximum allowed time lag of 10 ns along the WT and ME sequences for DNA alone and in complex with either CEBPB/E2F1-DP1 dimers, or with both TFs dimers CEBPB-E2F1-DP1. Correlation coefficients are calculated for the entire (0.1–1.1 μs) trajectories. In all panels, the E2F1-DP1 response element is marked with yellow, the CEBPB response element – with magenta. The maximum correlation coefficients (the correlations between the neighbouring b.p. steps were omitted from) with and without lag, along with the corresponding lag in ps is presented in the table below.

**B.p. step shift**<br>System	Maximum correlation without lag	Maximum correlation with lag	Time lag for maximum time-delayed correlation (ps)
WT DNA	-0.2316	-0.2334	-1
WT CEBPB	-0.2389	-0.2029	-76
WT E2F1-DP1	-0.2865	-0.2891	648
WT E2F1-DP1-CEBPB	-0.3737	-0.2687	-9999
ME DNA	0.1194	-0.1143	503
ME CEBPB	0.1745	0.1727	-261
ME E2F1-DP1	0.3411	0.3452	8303
ME E2F1-DP1-CEBPB	0.3744	0.3743	-55

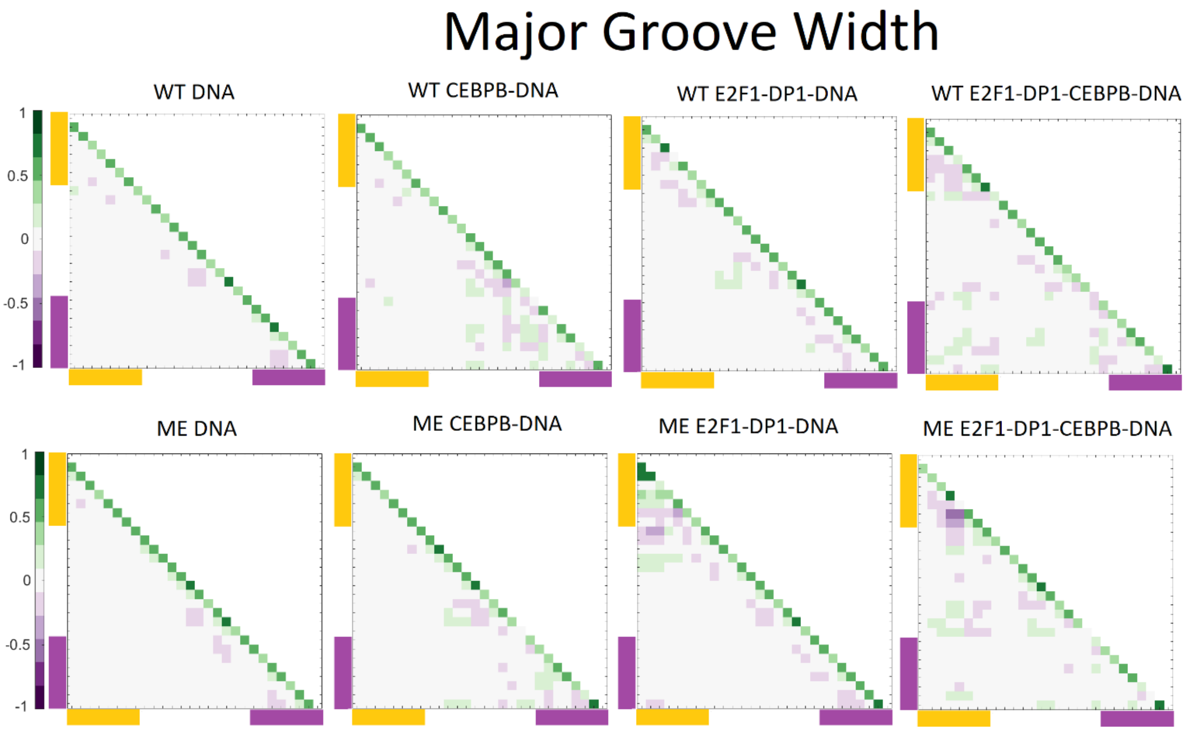

Figure S29: Time-delayed correlation coefficient maps for the major groove width, with a maximum allowed time lag of 10 ns along the WT and ME sequences for DNA alone and in complex with either CEBPB/E2F1-DP1 dimers, or with both TFs dimers CEBPB-E2F1-DP1. Correlation coefficients are calculated for the entire (0.1–1.1 μs) trajectories. In all panels, the E2F1-DP1 response element is marked with yellow, the CEBPB response element – with magenta. The maximum correlation coefficients (the correlations between the neighbouring b.p. steps were omitted from) with and without lag, along with the corresponding lag in ps is presented in the table below.

**Major groove width** System	Maximum correlation without lag	Maximum correlation with lag	Time lag for maximum correlation (ps)
WT DNA	-0.2672	-0.1737	-332
WT CEBPB	0.2583	-0.2577	-1
WT E2F1-DP1	-0.2543	-0.2369	271
WT E2F1-DP1-CEBPB	-0.2792	0.267	0
ME DNA	-0.2776	-0.1995	-511
ME CEBPB	-0.2421	-0.2095	-237
ME E2F1-DP1	-0.3375	-0.3174	89
ME E2F1-DP1-CEBPB	-0.5765	-0.5687	-26

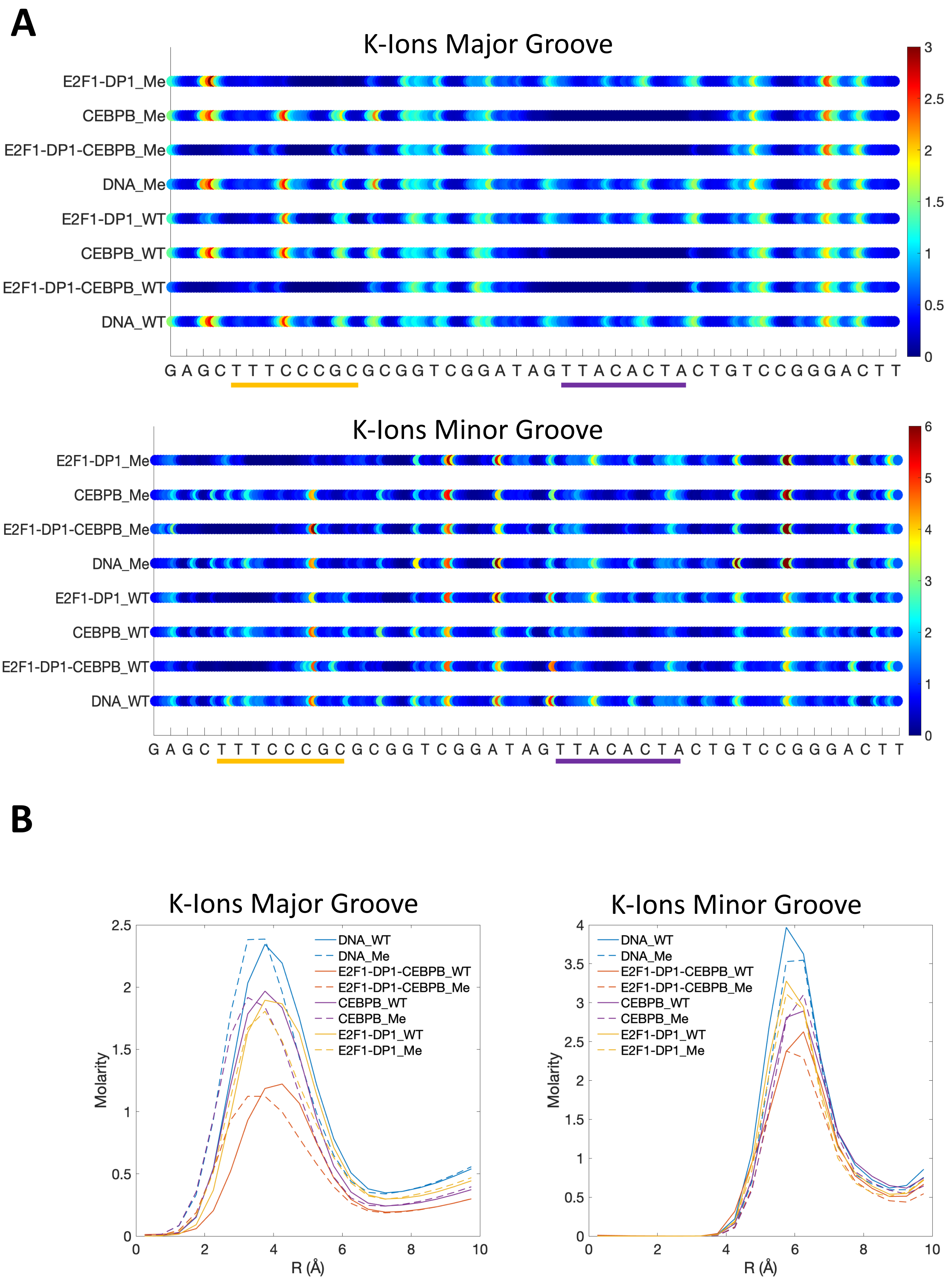

Figure S30: K+-molarity in major and minor groove (angles 147-33° for major groove and 33-147° for minor groove according to the settings in Canion (4)): **A.** along the DNA sequence, **B.** distance from the helical axis.

References:

1. Meissner A, Mikkelsen TS, Gu H, Wernig M, Hanna J, Sivachenko A, Zhang X, Bernstein BE, Nusbaum C, Jaffe DB et al. (2008) Genome-scale DNA methylation maps of pluripotent and differentiated cells. *Nature*. **454**, 766-770.

2. Liebl,K. and Zacharias,M. (2021) Accurate modeling of DNA conformational flexibility by a multivariate Ising model. *Proc. Natl. Acad. Sci.*, **118**, e2021263118.

3. Schlitter,J. (1993) Estimation of absolute and relative entropies of macromolecules using the covariance matrix. *Chem. Phys. Lett.*, **215**, 617–621.

4. Lavery,R., Moakher,M., Maddocks,J.H., Petkeviciute,D. and Zakrzewska,K. (2009) Conformational analysis of nucleic acids revisited: Curves+. *Nucleic Acids Res.*, **37**, 5917–5929.
